# Spin Manipulation of Co sites in Co_9_S_8_/Nb_2_CT_x_ Mott–Schottky Heterojunction for Boosting the Electrocatalytic Nitrogen Reduction Reaction

**DOI:** 10.1002/advs.202407301

**Published:** 2024-09-03

**Authors:** Shuai Zhang, Weihua Zhao, Jiameng Liu, Zheng Tao, Yinpeng Zhang, Shuangrun Zhao, Zhihong Zhang, Miao Du

**Affiliations:** ^1^ College of Material and Chemical Engineering Institute of New Energy Science and Technology School of Future Hydrogen Energy Technology Zhengzhou University of Light Industry Zhengzhou 450001 P. R. China; ^2^ School of Medical Engineering Xinxiang Medical University Xinxiang 453003 P. R. China

**Keywords:** ammonia synthesis, cobalt sulfide, Nb_2_CT_x_ MXene, nitrogen reduction reaction, spin manipulation

## Abstract

Regulating the adsorption of an intermediate on an electrocatalyst by manipulating the electron spin state of the transition metal is of great significance for promoting the activation of inert nitrogen molecules (N_2_) during the electrocatalytic nitrogen reduction reaction (eNRR). However, achieving this remains challenging. Herein, a novel 2D/2D Mott–Schottky heterojunction, Co_9_S_8_/Nb_2_CT_x_‐P, is developed as an eNRR catalyst. This is achieved through the in situ growth of cobalt sulfide (Co_9_S_8_) nanosheets over a Nb_2_CT_x_ MXene using a solution plasma modification method. Transformation of the Co spin state from low (t_2g_^6^e_g_^1^) to high (t_2g_^5^e_g_^2^) is achieved by adjusting the interface electronic structure and sulfur vacancy of Co_9_S_8_/Nb_2_CT_x_‐P. The adsorption ability of N_2_ is optimized through high spin Co(II) with more unpaired electrons, significantly accelerating the *N_2_→*NNH kinetic process. The Co_9_S_8_/Nb_2_CT_x_‐P exhibits a high NH_3_ yield of 62.62 µg h^−1^ mg_cat._^−1^ and a Faradaic efficiency (FE) of 30.33% at −0.40 V versus the reversible hydrogen electrode (RHE) in 0.1 m HCl. Additionally, it achieves an NH_3_ yield of 41.47 µg h^−1^ mg_cat._^−1^ and FE of 23.19% at −0.60 V versus RHE in 0.1 m Na_2_SO_4_. This work demonstrates a promising strategy for constructing heterojunction electrocatalysts for efficient eNRR.

## Graphical abstract

A novel 2D/2D Mott–Schottky heterojunction, Co_9_S_8_/Nb_2_CT_x_‐P is preparaed by a solution plasma modification method. The transformation of the Co 3*d* orbital electron configuration frow low (t_2g_^6^e_g_^1^) to high (t_2g_^5^e_g_^2^) spin is induced by S_v_ and interface effects. The highly spin‐polarized state of the Co_9_S_8_/Nb_2_CT_x_‐P demonstrated superior eNRR performance.

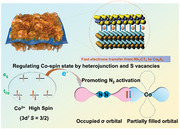

## 1 Introduction

As an excellent alternative to fossil fuels, ammonia (NH_3_) is regarded as a clean energy, offering advantages including low cost, easy availability, volatility, convenient storage, low pollution, high burning value, a high‐octane number, relatively safe operation, and compatibility with common materials.^[^ ^1^ ^]^ Despite the high N_2_ content in air (78%), converting N_2_ from air to NH_3_ and related compounds is challenging, owing to the inertness and high dissociation energy of molecular N_2_.^[^ ^2^ ^]^ Commercial NH_3_ is synthesized using the Haber–Bosch process, which requires harsh conditions, thereby leading to substantial energy consumption and significant CO_2_ emissions.^[^ ^3^ ^]^ In contrast to the traditional high‐energy Haber‐Bosch process, the electrocatalytic nitrogen reduction reaction (eNRR) offers an emerging alternative strategy for NH_3_ generation under environmental conditions, using renewable energy sources.^[^ ^4^ ^]^ However, the eNRR is typically conducted in acidic media. This is because the presence of abundant hydrogen protons (H^+^) promotes the formation of the *NNH intermediate, which is the rate‐determining step (RDS) in NH_3_ synthesis.^[^ ^5^ ^]^ Given the growing environmental concerns, there is a high demand for the conversion of N_2_ into NH_3_ in neutral media. Therefore, the development of advanced NRR electrocatalysts for the production of NH_3_ in pH‐universal media is highly desirable.

Transition metal atoms comprise incompletely filled *d* orbitals that can effectively activate N_2_. Consequently, various transition metal materials have been developed as effective catalysts for the eNRR.^[^ ^6^ ^]^ Nevertheless, the high electronegativity between the transition metal sites and neighboring N atoms renders the free energy of the intermediate (e.g., *NNH) unsuitable for adsorption.^[^ ^7^ ^]^ Electrocatalysts in a highly spin‐polarized state can promote N_2_ adsorption in the eNRR and regulate the bonding strength of the adsorption/desorption intermediates on the electrocatalyst surface, affording tunable surface electrochemical reaction kinetics.^[^ ^8^ ^]^ Transition metal spin states are closely related to the occupancy of the e_g_ and t_2g_ orbitals. Therefore, adjusting the electron configuration of these two orbitals can accelerate the eNRR and improve NH_3_ production efficiency. Various strategies for heteroatom doping,^[^ ^7^, ^9^ ^]^ element (e.g., S, O, N, or metal) vacancy engineering,^[^ ^10^ ^]^ and interface engineering^[^ ^11^ ^]^ have been exploited to regulate the electron configuration of the e_g_ and t_2g_ orbitals of active metal sites. For example, oxygen vacancies have been introduced into metal oxides (e.g., oxygen‐rich MoO_2_^[^ ^12^ ^]^ and TiO_2_^[^ ^13^ ^]^) to regulate the electronic state and adsorption properties of the active sites. This strategy weakens the N─N bond, thereby facilitating NH_3_ production.^[^ ^14^ ^]^ In addition, NH_3_ production using heterojunction electrocatalysts can be enhanced through the accumulation of reactants, formation of specific intermediates,^[^ ^15^ ^]^ and inhibition of byproducts.^[^ ^16^ ^]^ Current investigations on the spin states of active metal sites in heterojunctions mainly focus on revealing the origin of intrinsic activities.^[^ ^16^, ^17^ ^]^ However, research exploring the working mechanism of the electron spin states in such electrocatalysts and their impact on effective spin‐related electron transfer during the eNRR remains scarce. In particular, regulating the bonding strength of intermediates on electrocatalysts by manipulating the electron spin states of metal atomic active sites remains a significant challenge for promoting the activation of inert N_2_ during the eNRR.

In this study, a novel S‐vacancy (S_v_)‐enriched Co_9_S_8_ and Nb_2_CT_x_ MXene (T_x_ = surface terminal group) Mott–Schottky (M–S) heterojunction, Co_9_S_8_/Nb_2_CT_x_‐P, was prepared in situ using a solution plasma (SP) modification method for the efficient NH_3_ synthesis via the eNRR in acid and neutral electrolyte. During the SP irradiation procedure, a large number of free radicals and high‐energy particles can be generated, which further attack the electrocatalyst surface.^[^ ^18^ ^]^ Rich lattice defects and element vacancies thus formed on the electrocatalyst surface thanks to the dissociation of chemical bonds of active component, which enhanced the electron delocalization and spin state transition of the Co active sites. The highly spin‐polarized state stimulated the production of more unpaired electrons in the *d* orbitals of the Co sites, which effectively promoted the adsorption and activation of N_2_. Moreover, owing to the M–S effect, the strong electron coupling between the resulting heterojunctions further reduced the energy barrier for N_2_ protonation. Density functional theory (DFT) calculations revealed that the upward shift of the $d_{x^{2}}-d_{y^{2}}$ band center of the Co sites in Co_9_S_8_/Nb_2_CT_x_‐P energetically favored N_2_ adsorption and N─H bond formation in *NNH. The constructed S_v_‐enriched Co_9_S_8_/Nb_2_CT_x_‐P M–S heterojunction exhibited superior eNRR performances, yielding a high NH_3_ yield of 62.62 µg h^−1^ mg_cat._^−1^ at −0.40 V versus the reversible hydrogen electrode (RHE) in 0.1 m HCl, with a Faradaic efficiency (FE) of 30.33%. Additionally, it achieved an NH_3_ yield of 41.47 µg h^−1^ mg_cat._^−1^ at −0.60 V versus RHE in 0.1 m Na_2_SO_4_, with an FE of 23.19%. This work systematically confirms the application of spin engineering to eNRR activity, paving the way for the design of a new type of heterojunction electrocatalyst for the eNRR.

## 2 Results and Discussion

**Figure 1:**
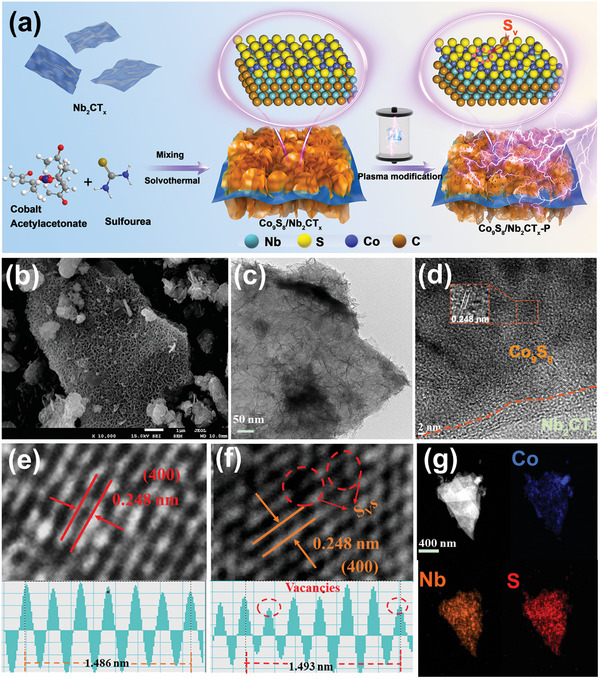
a) Schematic illustration of the preparation procedure of the Co_9_S_8_/Nb_2_CT_x_‐P M–S heterojunction. b) SEM, c) TEM, d) HR‐TEM images of the Co_9_S_8_/Nb_2_CT_x_‐P. e) HR‐TEM images and corresponding lattice line scanning of Co_9_S_8_/Nb_2_CT_x_, f) HR‐TEM images and corresponding lattice line scanning of Co_9_S_8_/Nb_2_CT_x_‐P. g) The EDS mapping of Co_9_S_8_/Nb_2_CT_x_‐P (blue: Co, orange: Nb, and red: S).

**Figure** **1**a illustrates the formation process of the Co_9_S_8_/Nb_2_CT_x_‐P M–S heterojunction. First the Co_9_S_8_/Nb_2_CT_x_ M–S heterojunction was constructed through the in situ growth of Co_9_S_8_ nanosheets (NSs) around Nb_2_CT_x_ MXene nanoflakes using a hydrothermal method. Subsequently, the obtained heterojunction was modified by SP treatment to form the Co_9_S_8_/Nb_2_CT_x_‐P M–S heterojunction comprising abundant S_v_s. Figure S1a,b (Supporting Information) clearly depicts that in the Co_9_S_8_/Nb_2_CT_x_, small‐sized NSs were vertically grown on the multilayered nanoflakes. Further, the edge of Co_9_S_8_/Nb_2_CT_x_ was identified as Co_9_S_8_NSs, which is marked with red dashed lines in the TEM image (Figure S1b, Supporting Information). The high‐resolution transmission electron microscopy (HR‐TEM) image of the Co_9_S_8_/Nb_2_CT_x_ (Figure S1c, Supporting Information) shows the connected structure of Co_9_S_8_ and Nb_2_CT_x_, along with a clear lattice spacing of 0.248 nm attributed to the (400) plane of Co_9_S_8_.

Similar results were observed for the heterojunction following SP treatment (Figure 1b–d). However, a few S_v_s were observed in the HR‐TEM image of the Co_9_S_8_/Nb_2_CT_x_‐P (Figure 1f, encircled in red), which were absent in that of the pristine heterojunction (Figure 1e). Additionally, the energy‐dispersive X‐ray spectroscopy mapping images of the Co_9_S_8_/Nb_2_CT_x_ and Co_9_S_8_/Nb_2_CT_x_‐P (Figure S1d, Supporting Information; Figure 1g, respectively) revealed the co‐existence and homogeneous distribution of Nb, Co, S, C, and O throughout the selected region, indicating the full coverage of Nb_2_CT_x_ with Co_9_S_8_ NSs. For comparison, the individual components, Co_9_S_8_ and Nb_2_CT_x_, before and after SP modification were also prepared, and their surface morphologies and nanostructures were analyzed (Figures S2 and S3, respectively). The Co_9_S_8_/Nb_2_CT_x_ demonstrated a larger Brunauer–Emmett–Teller surface area of 80.1 m^2^ g^−1^ compared to those of Co_9_S_8_ (51.9 m^2^ g^−1^) and Nb_2_CT_x_ (24.7 m^2^ g^−1^) (Figure S4a and Table S1, Supporting Information). This superior result is conducive to the penetration of the electrolyte and sufficient contact between the reactants and catalyst.

Figure S5 (Supporting Information) depicts the X‐ray diffraction (XRD) patterns of the Co_9_S_8_/Nb_2_CT_x_ M–S heterojunction before and after SP modification, revealing low crystallinity indicated by weak signals. Specifically, three weak diffraction peaks at 2θ = 29.7°, 47.8°, and 52.0° were observed in the XRD pattern of the heterojunction, corresponding to the (311), (511), and (440) facets of Co_9_S_8_, respectively. Furthermore, the diffraction peak at 2θ = 7.7°, which was attributed to the (002) plane of Nb_2_CT_x_, was relatively weak due to coverage by the Co_9_S_8_ NSs.

The Fourier‐transform infrared (FT‐IR) spectra of the heterojunction before and after the SP treatment were similar (Figure S6, Supporting Information). The characteristic band at 613 cm^−1^ was assigned to the stretching vibration of Co‐S bond in Co_9_S_8_. The peaks located at 483, 1107, 1363, and 1734 cm^−1^ were assigned to the Nb─C stretching, C─F, C─H, and C═O, respectively.

**Figure 2:**
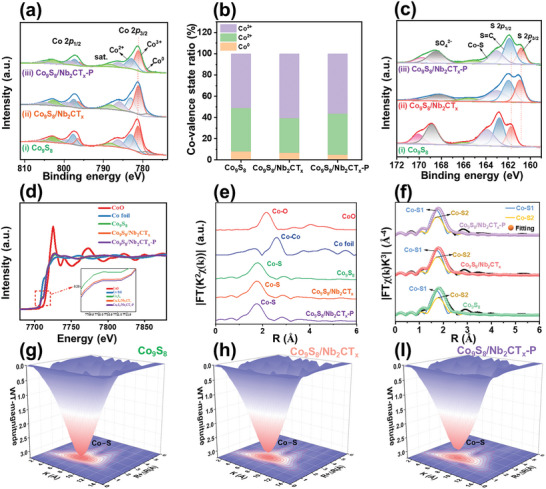
a) The Co 2*p* XPS spectra, and b) corresponding the different Co valence intensity ratio, c) S 2*p* XPS spectra of i) Co_9_S_8_, ii) Co_9_S_8_/Nb_2_CT_x_ and iii) Co_9_S_8_/Nb_2_CT_x_‐P. d) Co K‐edge XANES and e) FT‐EXAFS spectra of Co foil, CoO, Co_9_S_8_, Co_9_S_8_/Nb_2_CT_x_, and Co_9_S_8_/Nb_2_CT_x_‐P. f) FT‐EXAFS fitting results of Co_9_S_8_, Co_9_S_8_/Nb_2_CT_x_, and Co_9_S_8_/Nb_2_CT_x_‐P. WT‐EXAFS contour map of g) Co_9_S_8_, h) Co_9_S_8_/Nb_2_CT_x_, and I) Co_9_S_8_/Nb_2_CT_x_‐P.

The X‐ray photoelectron spectroscopy (XPS) survey scan spectra of the Co_9_S_8_/Nb_2_CT_x_ and Co_9_S_8_/Nb_2_CT_x_‐P (Figure S7, Supporting Information) displayed clear Nb 3*d* (207.3 eV), Co 2*p* (780.7 eV), S 2*p* (162.7 eV), C 1*s* (284.1 eV), and O 1*s* (531.6 eV) signals, further verifying the combination of the elemental signals of Co_9_S_8_ and Nb_2_CT_x_. The Co 2*p* XPS spectrum of Co_9_S_8_/Nb_2_CT_x_‐P (**Figure** **2**a) was deconvoluted into two pairs of peaks corresponding to Co 2*p*_3/2_ (781.1 eV) and Co 2*p*_1/2_ (797.1 eV). The pairs comprised peaks attributed to Co^0^ (779.2 and 796.2 eV), Co^3+^ (781.1 and 797.3 eV), and Co^2+^ (782.9 and 798.7 eV), indicating Co^0^/Co^2+^/Co^3+^mixed valence states. Notably, the binding energy (BE) positions of the Co 2*p*_3/2_ (781.3 eV) and Co 2*p*_1/2_ (797.1 eV) peaks of Co_9_S_8_/Nb_2_CT_x_‐P shifted negatively, by 0.2 and 0.4 eV, relative to those of the Co_9_S_8_/Nb_2_CT_x_ (781.1 and 797.5 eV), respectively. The peak positions of Co 2*p*_3/2_ (781.3 eV) and Co 2*p*_1/2_ (797.1 eV) in the Co_9_S_8_/Nb_2_CT_x_ spectrum (curve *ii*, Figure 2a) also exhibited a slight negative shift of 0.22 eV, compared to those of pristine Co_9_S_8_ (curve *i*, Figure 2a). Specifically, the electrons of the Co atom in Co_9_S_8_/Nb_2_CT_x_ can be transferred to the surrounding coordination environment.^[^ ^19^ ^]^ Moreover, the Co^2+^/Co^3+^ intensity ratio of the Co_9_S_8_/Nb_2_CT_x_‐P M–S heterojunction (0.69) was higher than that of pristine Co_9_S_8_/Nb_2_CT_x_ (0.54), and both were lower than that of Co_9_S_8_ (0.81) (Figure 2b).

During plasma irradiation in water, a high flux of hydrogen radicals, reactive oxygen species, electrons, and ions is produced via water splitting at the open end of the discharge zone.^[^ ^20^ ^]^ These active sites can then attack the Co─S bond in Co_9_S_8_, leading to its dissociation. Consequently, Co_9_S_8_/Nb_2_CT_x_‐P remains enriched with S_v_s with very low energy at the electrocatalyst surface, without altering the bulk properties. The pre‐occupied S 2*p* orbital electrons become delocalized around the Co^3+^ ions neighboring the S vacancies. Subsequently, the unpaired electrons resulting from the removal of S atoms can be accommodated in the orbitals of Co^3+^ ions, leading to the partial reduction of Co^3+^ to Co^2+^.^[^ ^21^ ^]^ Additionally, the S 2*p* XPS spectrum of Co_9_S_8_/Nb_2_CT_x_‐P (curve *iii*, Figure 2c) included peaks corresponding to Co─S (163.9 eV), C═S (162.7 eV), and S─O (168.9 eV). The peak positions of S 2*p*_3/2_ (160.7 eV) and S 2*p*_1/2_ (161.9 eV) in the Co_9_S_8_/Nb_2_CT_x_ spectrum (curve *ii*, Figure 2c) were negatively shifted by 0.7 and 0.8 eV compared to those of Co_9_S_8_ (161.7 and 162.8 eV) (curve *i*, Figure 2c), respectively. The M–S junction interface generated between Co_9_S_8_ and Nb_2_CT_x_ can modulate the density of Co active sites, resulting in electron transfer from Nb_2_CT_x_ to Co_9_S_8_.^[^ ^22^ ^]^ Moreover, the BE positions of S 2*p*_3/2_ and S 2*p*_1/2_ in the Co_9_S_8_/Nb_2_CT_x_‐P spectrum exhibited negative shifts of 0.2 and 0.12 eV, respectively, relative to those of the Co_9_S_8_/Nb_2_CT_x_ spectrum. This was attributed to the generation of abundant S_v_s in Co_9_S_8_/Nb_2_CT_x_‐P, which reduces the electron density on the S atom. In addition, the atomic composition, determined through XPS analysis, revealed a smaller S/Co atomic ratio of Co_9_S_8_/Nb_2_CT_x_‐P (0.53) compared to those of Co_9_S_8_ (0.69) and Co_9_S_8_/Nb_2_CT_x_ (0.62; Figures S8–S10, Supporting Information), indicating the formation of more S_v_s.

To further probe the local coordination geometry at the atomic level of Co_9_S_8_/Nb_2_CT_x_ before and after SP treatment, X‐ray absorption near‐edge structure (XANES) and extended X‐ray absorption fine structure (EXAFS) analyses were conducted on Co_9_S_8_, Co foil, and CoO for comparison. The Co_9_S_8_, Co_9_S_8_/Nb_2_CT_x_, and Co_9_S_8_/Nb_2_CT_x_‐P XANES spectra (Figure 2d) displayed similar peak shapes and adsorption edges, indicating their close valence states. Moreover, the energy absorption threshold of Co_9_S_8_/Nb_2_CT_x_‐P fell between those of the standard Co foil and CoO. These results indicated that the valence state of the Co atom in Co_9_S_8_/Nb_2_CT_x_‐P is higher than that of Co^0^ but lower than that of Co^2+^. The FT k^2^‐weighted phase‐uncorrected Co K‐edge EXAFS spectrum of Co_9_S_8_/Nb_2_CT_x_‐P (Figure 2e) displayed its main peak at 1.75 Å, attributed to the single scattering path of Co─S. Furthermore, the pristine heterojunction demonstrated a similar single scattering path of Co─S, which, however, was slightly shorter than that of Co_9_S_8_ (1.79 Å). Notably, the peak intensity of the Co─S bond in Co_9_S_8_/Nb_2_CT_x_‐P was lower than those in Co_9_S_8_/Nb_2_CT_x_ and Co_9_S_8_. This weaker Co─S peak intensity of Co_9_S_8_/Nb_2_CT_x_‐P was primarily attributed to the presence of S_v_s.

The quantitative least‐squares EXAFS curves were fitted to analyze the local coordination of Co in the three catalysts (Figures 2f and S11 and Table S2, Supporting Information). Co_9_S_8_ presented two different sets, namely Co─S1 with bond length of 2.08 Å and Co─S2 with bond length of 2.21 Å, suggesting the coexistence of two different unit cells. The coordination numbers for Co─S1 and Co─S2 were fitted to be 1.53 and 3.19, respectively. For the Co_9_S_8_/Nb_2_CT_x_‐P heterojunction, the coordination number for Co─S1 was determined to be 1.26, which is lower than those of Co_9_S_8_ (1.53) and Co_9_S_8_/Nb_2_CT_x_ (1.47) owing to the abundant formation of S_v_s. The decreased length of the Co─S bond and the low coordination number of Co_9_S_8_/Nb_2_CT_x_‐P demonstrate negligible structural deformation attributed to the high population of S_v_s. Moreover, the wavelet transform analysis of the three samples (Figure 2g–I) revealed that the Co─S path exhibited maximum intensity at *R* ≈ 2.15 Å and *k* ≈ 6.7 Å^−1^.

**Figure 3:**
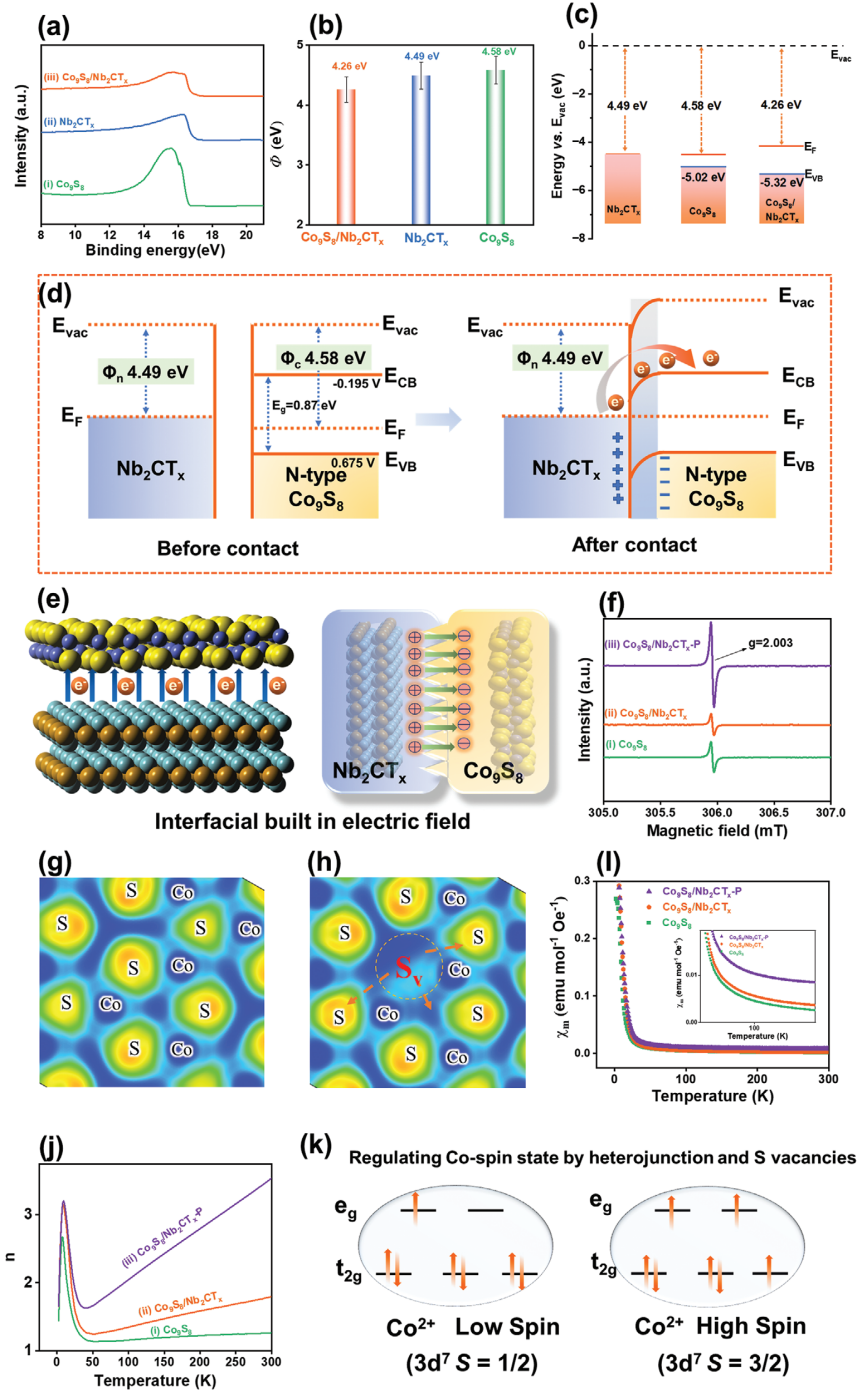
a) UPS spectra in the normalized secondary electron cutoff energy (*E*_cutoff_) regions of i) Co_9_S_8_, ii) Nb_2_CT_x_, and iii) Co_9_S_8_/Nb_2_CT_x_. b) Calculated *Φ* stemming from UPS spectra. c) Energy band structures of Co_9_S_8_, Nb_2_CT_x_, and Co_9_S_8_/Nb_2_CT_x_ (E_F_: Fermi level, E_VB_: valence band level, and E_vac_: vacuum level). d) Energy band diagrams of Nb_2_CT_x_ and Co_9_S_8_ before and after the Mott‐Schottky junction formation. e) Schematic illustration of the interfacial electronic couple effect between Nb_2_CT_x_ and Co_9_S_8_. f) EPR spectra i) Co_9_S_8_, ii) Co_9_S_8_/Nb_2_CT_x_, iii) Co_9_S_8_/Nb_2_CT_x_‐P. The electron localization function of g) Co_9_S_8_/Nb_2_CT_x_ and h) Co_9_S_8_/Nb_2_CT_x_‐P. I) Magnetic susceptibilities and j) The number of unpaired electrons of i) Co_9_S_8_, ii) Co_9_S_8_/Nb_2_CT_x_, iii) Co_9_S_8_/Nb_2_CT_x_‐P. k) Illustration of Co^2+^ spin states in Co_9_S_8_/Nb_2_CT_x_‐P.

The normalized secondary electron cutoff energies of the samples were measured using ultraviolet photoelectron spectroscopy (UPS) (**Figure** **3**a), enabling the deduction of the work function (*Φ*) of the electrocatalyst. As illustrated in Figure 3b, the *Φ* of Co_9_S_8_/Nb_2_CT_x_ is ≈4.26 eV, which is marginally smaller than those of Co_9_S_8_ (4.58 eV) and Nb_2_CT_x_ (4.49 eV). The semiconductor type of Co_9_S_8_ and Co_9_S_8_/Nb_2_CT_x_ was probed using M–S curves (Figure S12, Supporting Information), revealing characteristics consistent with *n*‐type semiconductors. Ultraviolet‐visible (UV–vis) absorption spectra were recorded for all the prepared electrocatalysts (Figure S12c, Supporting Information). Additionally, the forbidden bands of Nb_2_CT_x_, Co_9_S_8_, and Co_9_S_8_/Nb_2_CT_x_ were calculated as 0.84, 0.87, and 0.78 eV, respectively, from Tauc plots (Figure S12d–f, Supporting Information).

The combination of M–S plots, UPS, and UV–vis results confirmed the formation of an M–S heterojunction between Nb_2_CT_x_ and Co_9_S_8_. Furthermore, the energy band plots and band structures of Nb_2_CT_x_ and Co_9_S_8_ (Figure 3c,d) revealed the redistribution of electrons at the interface between Nb_2_CT_x_ and Co_9_S_8_ owing to the Schottky barrier. When Nb_2_CT_x_ and Co_9_S_8_ come into contact and form a heterojunction interface, electrons from Nb_2_CT_x_ spontaneously flow to Co_9_S_8_ until dynamic equilibrium is reached in terms of the Fermi levels. At this moment, the holes in the valence band of Co_9_S_8_ flow to Nb_2_CT_x_, facilitating charge transfer at the interface. This induces band bending and generates an internal electric field at the M–S interfaces, thereby promoting the flow of electrons from Nb_2_CT_x_ into Co_9_S_8_ (Figure 3e). In the presence of the Fermi level and interfacial built‐in electric field, interfacial electron transfer accelerates the catalytic process of the eNRR. This acceleration is attributed to the tendency of the S_v_‐enriched Co_9_S_8_/Nb_2_CT_x_‐P to donate more electrons to the intermediates in the eNRR process.

Theoretically, the eNRR performance of transition metal‐based electrocatalysts is related to the occupancy rates of their *d* orbitals.^[^ ^23^ ^]^ Therefore, to investigate the electronic configurations of the Co center, we conducted electron paramagnetic resonance (EPR), zero‐field cooling (ZFC) temperature‐dependent magnetization (χ_m_). Figure 3f illustrates the signals of the Co─S dangling bonds at *g* = 2.003 in the EPR spectra of Co_9_S_8_/Nb_2_CT_x_ before and after SP modification, which are proportional to the concentrations of dangling bonds from the S_v_s in Co_9_S_8_/Nb_2_CT_x_ and Co_9_S_8_/Nb_2_CT_x_‐P. The intensity of the *g* signal of Co_9_S_8_/Nb_2_CT_x_‐P was higher than that of the pristine heterojunction, indicating an increase in the number of defects. Specifically, the S_v_s in Co_9_S_8_/Nb_2_CT_x_‐P result from a region of electron delocalization at the S position, further causing a slight localization of electrons in the surrounding S atom (Figure 3g,h). Consequently, the electronic structure of the surrounding Co active site can be modulated, facilitating electron transfer from the catalyst to N_2_ and the intermediates generated in the eNRR process. This enhances the NRR performance of the constructed electrocatalyst.

Given that charge redistribution is accompanied by the transition of the 3*d* electron spin configuration, temperature‐dependent magnetization in the ZFC mode was conducted at 1000 Oe in the temperature range of 5–300 K to gain deeper insight into the effect of the magnetic moments of Co_9_S_8_, Co_9_S_8_/Nb_2_CT_x_, and Co_9_S_8_/Nb_2_CT_x_‐P. From the χ_m_ versus T plots (Figure 3I), the effective magnetic moment (µ_eff_) of Co_9_S_8_/Nb_2_CT_x_‐P was estimated to be 4.34 µ_B_, which is markedly higher than those of Co_9_S_8_ (2.03 µ_B_) and Co_9_S_8_/Nb_2_CT_x_ (2.01 µ_B_). Therefore, with an increase in the number of S_v_s, electrons can fill the high‐energy orbital (*e*_g_), thereby improving the spin state of Co. According to the equation 2.828$\sqrt{\chi_{m}T}$ = µ_eff_ = $\sqrt{n(n+2)\;},$ ^[^ ^24^ ^]^ the number of unpaired *d* electrons (*n*) in Co_9_S_8_/Nb_2_CT_x_‐P (Figure 3j) was calculated to be ≈3.45, which is significantly larger than those in Co_9_S_8_/Nb_2_CT_x_ (≈1.24) and Co_9_S_8_ (≈1.26). The result indicated that Co_9_S_8_/Nb_2_CT_x_‐P had three unpaired electrons, leading to a high spin state (t_2g_^5^e_g_^2^). However, Co_9_S_8_/Nb_2_CT_x_ and Co_9_S_8_ only had one unpaired electron, forming a low spin state (t_2g_^6^e_g_^1^). As a result, it determines the electronic structure of Co 3*d* in Co_9_S_8_/Nb_2_CT_x_‐P (Figure 3k).

**Figure 4:**
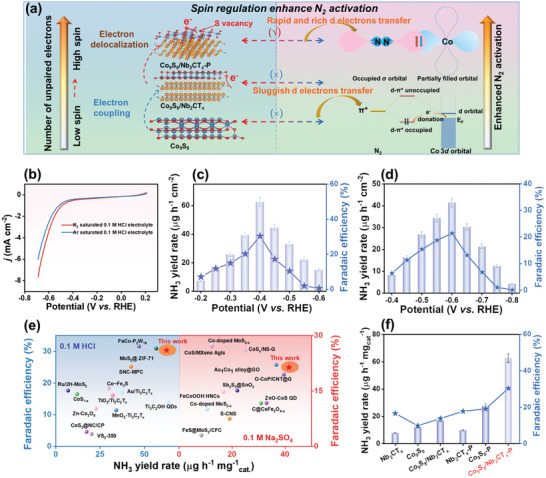
a) The schematic illustration of the spin regulation of Co 3*d* for the acceleration of the protonation process. b) LSV polarization curves of Co_9_S_8_/Nb_2_CT_x_‐P in an N_2_‐ and Ar‐saturated 0.1 m HCl electrolyte. Dependence of the NH_3_ yield and FE of Co_9_S_8_/Nb_2_CT_x_‐P at each applied potential in N_2_‐saturated c) 0.1 m HCl and d) 0.1 m Na_2_SO_4_ with the NRR measurement time of 6000 s. e) The eNRR performances of different electrocatalysts of Co_9_S_8_, Nb_2_CT_x_, and Co_9_S_8_/Nb_2_CT_x_ before and after the SP treatment at −0.40 V versus RHE in 0.1 m HCl. f) Comparison of the NH_3_ yield rate of Co_9_S_8_/Nb_2_CT_x_‐P with some reported electrocatalysts.

Owing to quantum spin exchange interactions, a greater number of unpaired electrons occupying the active orbital can enhance the catalytic activity of the electrocatalyst by weakening the binding of the reaction intermediates.^[^ ^25^ ^]^ Accordingly, the strong interaction between the S_v_s and the formation of the heterojunction effectively reshaped the electronic structure of Co, resulting in a Co 3*d* electron spin configuration transition from low to high. Thus, Co_9_S_8_/Nb_2_CT_x_‐P exhibited more unpaired electrons in the 3*d* orbitals, which enhanced its catalytic activity compared to those of Co_9_S_8_/Nb_2_CT_x_ and Co_9_S_8_ (**Figure** **4**a).

The primary product of eNRR is NH_3_, which can be captured by electrolyte solution. Additionally, N_2_H_2_ as an unexpected by‐product also affects the performance of eNRR. Therefore, the production of NH_3_ and N_2_H_4_ in the cathode compartment in each of the two solutions was measured after each electrolysis procedure, according to the calibration curves standardized by the indophenol blue and Watt–Chrisp method methods (Figures S13–S16, Supporting Information). The eNRR performance of the Co_9_S_8_/Nb_2_CT_x_‐P M–S heterojunction was investigated in both 0.1 m HCl and 0.1 m Na_2_SO_4_, using Co_9_S_8_ and pristine Co_9_S_8_/Nb_2_CT_x_ for comparison. Amongst, the influence of the Co_9_S_8_ content in Co_9_S_8_/Nb_2_CT_x_‐P on the NRR performances was systematically probed (Figure S17, Supporting Information). Linear sweep voltammetry polarization curves of Co_9_S_8_/Nb_2_CT_x_‐P were recorded separately in Ar‐ and N_2_‐saturated 0.1 m HCl (Figure 4b) and 0.1 m Na_2_SO_4_ (Figure S18, Supporting Information) at a scan rate of 5 mV s^−1^. A significant current density gap of Co_9_S_8_/Nb_2_CT_x_‐P was observed within the potential window of −0.23 to −0.70 V and −0.68 to −1.10 V versus RHE in 0.1 m HCl and 0.1 m Na_2_SO_4_, respectively. The onset potential of Co_9_S_8_/Nb_2_CT_x_‐P in 0.1 m HCl electrolyte is −0.40 V, while it in 0.1 m Na_2_SO_4_ is −0.68 V, in which the onset potential is determined at the current density of −0.5 mA cm^−2^. As results, the current gap of Co_9_S_8_/Nb_2_CT_x_‐P between N_2_ and Ar saturated electrolyte indicates that enhanced eNRR may occur at the nitrogen/electrocatalyst/electrolyte three‐phase interface. Additionally, cyclic voltammetry curves and Tafel plots of Co_9_S_8_/Nb_2_CT_x_‐P were investigated in Ar‐ and N_2_‐saturated 0.1 m HCl or Na_2_SO_4_ electrolyte (Figure S19, Supporting Information). Subsequently, chronoamperometry tests were conducted for 6000 s in 0.1 m HCl and 0.1 m Na_2_SO_4_ to evaluate the NH_3_ yield and FE of Co_9_S_8_/Nb_2_CT_x_‐P (Figure S20, Supporting Information). The NH_3_ yield was calculated from the UV–vis absorption spectra of the electrolyte (Figure S21, Supporting Information). Notably, no significant N_2_H_4_ byproduct was detected during the eNRR (Figure S22, Supporting Information). These results indicate that the Co_9_S_8_/Nb_2_CT_x_‐P M–S heterojunction exhibits good selectivity for NH_3_ production in both acidic and neutral media. As depicted in Figure 4c, Co_9_S_8_/Nb_2_CT_x_‐P exhibited a maximum NH_3_ yield rate of 62.62 µg h^−1^ mg_cat._^−1^ and a high FE of 30.33% at −0.40 V versus RHE in 0.1 m HCl. However, when a more negative potential was applied, both the NH_3_ yield and FE of Co_9_S_8_/Nb_2_CT_x_‐P decreased markedly, owing to the competing hydrogen evolution reaction (HER). In addition, the electrocatalyst displayed extraordinary eNRR activity in 0.1 m Na_2_SO_4_ (Figure 4d), achieving a maximum NH_3_ yield of 41.47 µg h^−1^ mg_cat._^−1^ at −0.60 V versus RHE and an FE of 23.19%.

As illustrated in Figure 4e, the NH_3_ yield and FE of the Co_9_S_8_/Nb_2_CT_x_‐P heterojunction are also higher than those of Co_9_S_8_ (NH_3_ yield, 7.85 µg h^−1^ mg_cat._^−1^; FE, 16.62%) and Co_9_S_8_/Nb_2_CT_x_ (NH_3_ yield, 16.89 µg h^−1^ mg_cat._^−1^; FE, 13.83%) at −0.40 V versus RHE in 0.1 HCl. This performance also surpasses those of other Co‐ or MXene‐based electrocatalysts (Table S3, Supporting Information; Figure 4f), such as Co‐SAs/NC,^[^ ^26^ ^]^ CoP_3_/CC,^[^ ^27^ ^]^ 1T‐MoS_2_/g‐C_3_N_4_,^[^ ^28^ ^]^ MnO_2_‐Ti_3_C_2_T_x_,^[^ ^29^ ^]^ and C@CoS@TiO_2_.^[^ ^30^ ^]^ Similarly, the NH_3_ production rate and FE of Co_9_S_8_/Nb_2_CT_x_‐P in a neutral medium exceed those of most state‐of‐art electrocatalysts under the same conditions (Figure 4f; Table S4, Supporting Information), such as ZnO‐CoS QD,^[^ ^31^ ^]^ FeCoOOH HNCs,^[^ ^32^ ^]^ O‐CoP/CNT@G,^[^ ^33^ ^]^ and CoS@S‐Mas.^[^ ^34^ ^]^ This highlights the potential application of the constructed Co_9_S_8_/Nb_2_CT_x_‐P electrocatalyst for NH_3_ production under ambient conditions. The superior eNRR ability of Co_9_S_8_/Nb_2_CT_x_‐P is ascribed to the strong electronic coupling of Co_9_S_8_ with Nb_2_CT_x_ and the abundant S_v_s, which significantly adjust the electronic structure of the Co active sites. The presence of more unpaired electrons in the 3*d* orbital of the Co active sites enhances the activation ability toward N_2_ molecules, thereby boosting the eNRR performance.

**Figure 5:**
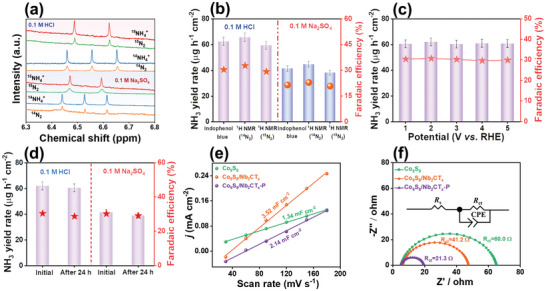
^15^N_2_, ^14^N_2_ isotope labeling and indophenol blue experiments in 0.1 m HCl and 0.1 m Na_2_SO_4_. a) Baseline‐subtracted ^1^H NMR spectra of the post‐electrolyte with ^15^N_2_ and ^14^N_2_ compared with the reference ^14^NH_4_Cl and ^15^NH_4_Cl. b) Comparison of the NH_3_ yields calculated by NMR and indophenol blue tests. c) Cycling stability of Co_9_S_8_/Nb_2_CT_x_‐P at −0.40 V versus RHE in 0.1 m HCl. d) The NH_3_ yield and FE of original and post‐eNRR electrolysis in 0.1 M HCl and 0.1 M Na_2_SO_4_. e) The corresponding electrochemical double layer capacitances and f) the EIS Nyquist plots of Co_9_S_8_, Co_9_S_8_/Nb_2_CT_x_, and Co_9_S_8_/Nb_2_CT_x_ ‐P at −0.4 V versus RHE in 0.1 m HCl. Inset: equivalent circuit model, where *R*_s_ is the electrolytic resistance, *R*_ct_ is charge‐transfer resistance, and CPE (constant phase angle element) is the double‐layer capacitance.

A control experiment was conducted under the same conditions using bare carbon paper (CP) at an open‐circuit voltage in N_2_‐saturated 0.1 m HCl (Figure S23a, Supporting Information) and 0.1 m Na_2_SO_4_ (Figure S23b Supporting Information). However, no significant NH_3_ production was observed with bare CP, indicating that NH_3_ is generated entirely from the eNRR catalyzed by Co_9_S_8_/Nb_2_CT_x_‐P. The origin of the N source was further determined using isotopic labeling measurements. As illustrated in the NMR spectra in Figures **5**a and S24–S27 (Supporting Information), a distinct triplet (or doublet) coupling of ^14^NH_4_^+^ (or ^15^NH_4_^+^) was observed when ^14^N_2_ (or ^15^N_2_) was used as the feeding gas. In contrast, no significant ^14^NH_4_^+^ (or ^15^NH_4_^+^) signals were detected when the feeding gas was Ar (Figure S28, Supporting Information). The corresponding yields of ^14^N‐ and ^15^N‐labeled NH_3_ obtained using ^1^H NMR show that the amounts of NH_3_ produced by Co_9_S_8_/Nb_2_CT_x_‐P in 0.1 m HCl were 65.75 and 59.39 µg h^−1^ mg_cat._^−1^, respectively. In 0.1 m Na_2_SO_4_, these values decreased to 44.74 and 38.29 µg h^−1^ mg_cat._^−1^, respectively. These results are similar to those obtained using the indophenol blue method (Figure 5b). The introduced gas and experimental environment were carefully controlled throughout the experiments. Therefore, we supposed that the difference between the indophenol blue and isotopic labeling methods originated from experimental errors.

The stability of Co_9_S_8_/Nb_2_CT_x_‐P was assessed in 0.1 m HCl (Figure 5c) and 0.1 m Na_2_SO_4_ (Figure S29, Supporting Information). No substantial variations were observed in either the NH_3_ yields or FEs during five cycles of chronoamperometric runs, indicating the excellent stability of Co_9_S_8_/Nb_2_CT_x_‐P. Moreover, the chronoamperometry curves indicated that the current density of Co_9_S_8_/Nb_2_CT_x_‐P toward the eNRR remained stable without significant fluctuation, for 24 h, in both 0.1 m HCl (Figure S30a, Supporting Information) and 0.1 m Na_2_SO_4_ (Figure S30b, Supporting Information). Consequently, no significant changes were observed in the NH_3_ production rate or FE before and after 24 h (Figure 5d), verifying the high stability of Co_9_S_8_/Nb_2_CT_x_‐P toward the eNRR. In addition, the pH value of the post‐electrolyte after eNRR was studied, revealing a negligible difference in pH between the 0.1 m HCl and 0.1 m Na_2_SO_4_ electrolytes (Figure S31, Supporting Information). The SEM and TEM images and XRD patterns of Co_9_S_8_/Nb_2_CT_x_‐P after the long‐term tests in 0.1 m HCl (Figure S32, Supporting Information) and 0.1 m Na_2_SO_4_ (Figure S33, Supporting Information) also displayed no substantial changes, further confirming its good structural stability. The XPS characterizations of the spent electrocatalyst showed that the BE positions of the Co 2*p* (Figure S34a, Supporting Information) and S 2*p* XPS (Figure S34b, Supporting Information) spectra displayed positive shifts in both 0.1 m HCl and 0.1 m Na_2_SO_4_. This indicated that the electron density of the Co active sites in Co_9_S_8_ increased after use in the eNRR. In contrast, the Nb 3*d* XPS spectrum (Figure S34c, Supporting Information) displayed a negative shift, revealing a decrease in the electron density of the Nb sites. Collectively, these observations suggest that electron transfer occurred between Co_9_S_8_ and Nb_2_CT_x_, indicating charge reconstruction during the eNRR process.

To elucidate the catalytic mechanism of the eNRR, we conducted electrochemical active surface area and electrochemical impedance spectroscopy (EIS) measurements for all the catalysts in 0.1 m HCl and 0.1 m Na_2_SO_4_. Cyclic voltammetry curves of all samples (Figure S35, Supporting Information), obtained within the narrow potential window of 0.10–0.20 V versus Ag/AgCl (saturated KCl solution), were constructed to evaluate the double‐layer capacitance (*C*_dl_), which represents the electrochemical surface area. Co_9_S_8_/Nb_2_CT_x_‐P displayed a *C*_dl_ value of 2.14 mF cm^−2^, which was higher than that of Co_9_S_8_ (1.34 mF cm^−2^) and lower than that of Co_9_S_8_/Nb_2_CT_x_ (3.52 mF cm^−2^) (Figure 5e). Moreover, the EIS Nyquist plots (Figure 5f) revealed that Co_9_S_8_/Nb_2_CT_x_‐P exhibited a smaller *R*_ct_ (21.3 Ω) compared to those of Co_9_S_8_ (41.2 Ω) and Co_9_S_8_/Nb_2_CT_x_ (60.0 Ω). In 0.1 m Na_2_SO_4_, the *C*_dl_ of Co_9_S_8_/Nb_2_CT_x_‐P (4.20 mF cm^−2^) was slightly higher than that of Co_9_S_8_/Nb_2_CT_x_ (3.60 mF cm^−2^) (Figure S36, Supporting Information). Similarly, the EIS Nyquist plot of Co_9_S_8_/Nb_2_CT_x_‐P in 0.1 m Na_2_SO_4_ (Figure S37, Supporting Information) indicated a smaller *R*_ct_ (54.6 Ω) compared to those of Co_9_S_8_ (110.0 Ω) and Co_9_S_8_/Nb_2_CT_x_ (71.2 Ω). These results suggest that Co_9_S_8_/Nb_2_CT_x_‐P facilitates faster electron transfer and possesses more catalytically active sites than the other catalysts, thereby enhancing the eNRR performance at a relatively low overpotential.

**Figure 6:**
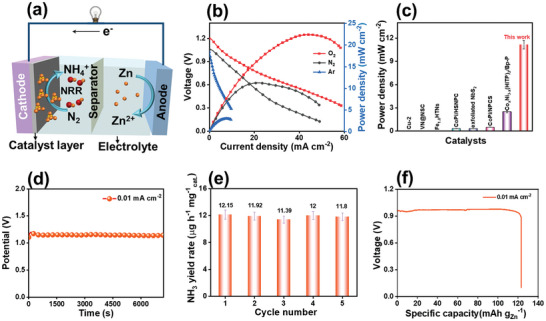
a) Schematic diagram of a rechargeable Zn–N_2_ battery. b) Discharging polarization curves and the power density plot of the Co_9_S_8_/Nb_2_CT_x_‐P‐assembled Zn–N_2_ battery. c) Comparison of the power densities of the Zn–N_2_ battery assembled with different catalysts. d) The discharge curve of the constructed Zn–N_2_ battery with Co_9_S_8_/Nb_2_CT_x_‐P at 0.01 mA cm^−2^ for 7200 s. e) The NH_3_ yields produced by the Zn–N_2_ battery with Co_9_S_8_/Nb_2_CT_x_‐P for five repeated tests. f) The discharge curve of the manufactured Zn–N_2_ battery at the current density of 0.01 mA cm^−2^.

Given the superior eNRR ability demonstrated by the constructed Co_9_S_8_/Nb_2_CT_x_‐P M–S junction, assembly of a Zn–N_2_ aqueous battery for NH_3_ synthesis and simultaneous electricity generation is envisioned.^[^ ^35^ ^]^ To explore this possibility, we conceptually manufactured a home‐made Zn–N_2_ battery composed of a cathode of Co_9_S_8_/Nb_2_CT_x_‐P coated on the CP and an anode of Zn foil. A photograph of the reaction cell is shown in **Figure** **6**a. N_2_ was continuously bubbled into the cathode within an asymmetric electrolyte system comprising an acid catholyte and alkaline anolyte separated by a bipolar membrane.

The rate–discharge curve of Co_9_S_8_/Nb_2_CT_x_‐P in Figure 6b displays the voltage changes at different current densities. As intended, the Zn–N_2_ battery assembled with Co_9_S_8_/Nb_2_CT_x_‐P delivered a high discharge current density, peaking at 48.8 mA cm^−2^ at 0.10 V versus Zn^2+^/Zn. Furthermore, Figure 6b shows that the Co_9_S_8_/Nb_2_CT_x_‐P‐based Zn–N_2_ cell can deliver a power density of 11.17 mW cm^−2^, significantly outperforming some reported Zn–N_2_ batteries (Figure 6c), including Co_x_Ni_3‐x_(HITP)_2_/BNSs‐P (2.5 mW cm^−2^),^[^ ^36^ ^]^ CoPi/NPCS (0.49 mW cm^−2^),^[^ ^37^ ^]^ and CoPi/HSNPC (0.31 mW cm^−2^).^[^ ^38^ ^]^ After the constructed cell was discharged at 0.01 mA cm^−2^ for 7200 s (Figure 6d), an average NH_3_ yield of 11.8 µg h^−1^ cm^−2^ (Figure 6e) was determined based on five repeated tests. Additionally, a high energy density of 124 mA h g^−1^ was achieved at 0.01 mA cm^−2^ (Figure 6f).

**Figure 7:**
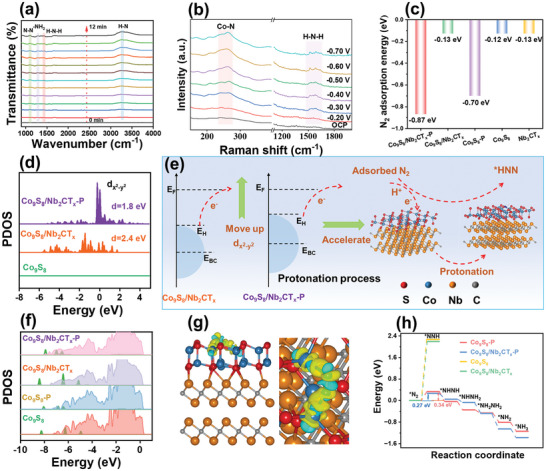
a) In situ electrochemical FT‐IR spectra and b) in situ Raman spectra on Co_9_S_8_/Nb_2_CT_x_‐P during the eNRR in 0.1 m HCl. c) The N_2_ adsorption energy of Nb_2_CT_x_, Co_9_S_8_, Co_9_S_8_‐P, Co_9_S_8_/Nb_2_CT_x_, and Co_9_S_8_/Nb_2_CT_x_‐P. d) The projected density of states of Co 3$d_{x^{2}}-d_{y^{2}}$ orbitals for Co_9_S_8_, Co_9_S_8_/Nb_2_CT_x_, and Co_9_S_8_/Nb_2_CT_x_‐P. e) The schematic illustration of the shift of $d_{x^{2}}-d_{y^{2}}$ orbital toward *E*_F_ on the facilitation of the protonation of Co_9_S_8_/Nb_2_CT_x_‐P, *E*_H_, and *E*_BC_ represent the $d_{x^{2}}-d_{y^{2}}$ highest occupied crystal orbital and band center, respectively. f) Partial density of states of N_2_ adsorption on Co_9_S_8_, Co_9_S_8_‐P, Co_9_S_8_/Nb_2_CT_x_, and Co_9_S_8_/Nb_2_CT_x_‐P. g) Charge density difference of N_2_ adsorption on Co_9_S_8_/Nb_2_CT_x_‐P (red sphere: S, blue sphere: Co, brown sphere: Nb, and gray sphere: C. Yellow and cyan‐colored iso‐surfaces show electron gain and loss, respectively). h) Calculated Gibbs free energies of the eNRR on Co_9_S_8_‐P, Co9S8, Co_9_S_8_/Nb_2_CT_x_, and Co_9_S_8_/Nb_2_CT_x_‐P along alternating pathways.

To thoroughly elucidate the electrocatalytic pathway of the eNRR on Co_9_S_8_/Nb_2_CT_x_‐P, time‐dependent in situ ATR‐FTIR spectra were recorded in 0.1 m N_2_‐saturated HCl at −0.40 V versus RHE. As depicted in **Figure** **7**a, the appearance of the absorption peak at 1104 cm^−1^, corresponding to the N─N tensile bond, suggests the dissociation of the N≡N bond of the adsorbed N_2_ molecules on the working electrode surface.^[^ ^39^ ^]^ Additionally, the three adsorption peaks at 1272, 1442, and 3278 cm^−1^ were assigned to ─NH_2_ vibration, H─N─H bending, and N─H stretching vibrations, respectively.^[^ ^40^ ^]^ Notably, the intensities of these peaks increased with increasing reaction time. Consequently, we supposed that the reaction intermediates accumulate during the eNRR process. Most importantly, the absorption peak at 1637 cm^−1^, attributed to N_2_ molecules adsorbed on the catalyst surface, gradually decreases in intensity, indicating the consumption of N_2_ molecules.^[^ ^36^ ^]^ These results suggest that the eNRR over the Co_9_S_8_/Nb_2_CT_x_‐P surface may follow an alternative pathway. Similar results were observed in the in situ ATR‐FTIR spectra of the electrocatalyst recorded in 0.1 m Na_2_SO_4_ (Figure S38, Supporting Information), indicating the same catalytic pathway.

To identify the operative active sites of Co_9_S_8_/Nb_2_CT_x_‐P, the eNRR was further monitored via electrochemical in situ Raman spectroscopy at various potentials (Figure 7b). Notable Co─N bond formation was observed at 263 cm^−1^ with increasing applied potential,^[^ ^41^ ^]^ indicating that N_2_ molecules were predominately captured by the Co site (Figure 7b). Furthermore, the significant peak at ≈1578.5 cm^−1^ was assigned to H─N─H tensile vibration (Figure 7b).^[^ ^42^ ^]^ Notably, the intensity of this peak decreased with increasing potential, possibly due to NH_3_ accumulation. The strongest Co─N bond was observed at −0.40 V versus RHE in the potential‐dependent in situ Raman spectra, consistent with experimental findings.

To further elucidate the activation process of N_2_ at the Co site and the evolution of intermediates during the eNRR process, DFT calculations were performed to provide deeper insight into the electrocatalytic mechanism of the prepared catalyst toward the NRR. As depicted in Figure 7c, the adsorption energy of N_2_ molecules on the Co_9_S_8_/Nb_2_CT_x_‐P surface was higher compared to that of the plasma‐modified Co_9_S_8_ (Co_9_S_8_‐P) and markedly higher than those of pristine Co_9_S_8_, Nb_2_CT_x_, and Co_9_S_8_/Nb_2_CT_x_. This finding reveals that the formation of a microinterface between the diverse components and the generation of rich S vacancies through plasma modification can enhance N_2_ adsorption, thereby improving the eNRR performance. In terms of the density of states (DOS) (Figure S39, Supporting Information), Co_9_S_8_/Nb_2_CT_x_‐P displayed an increased integral area of the net spin‐up compared to both Co_9_S_8_ and Co_9_S_8_/Nb_2_CT_x_. This observation confirms that the S_v_s and interface effects lead to an increase in the number of spin‐up electrons, which is consistent with the ZFC temperature‐dependent magnetic susceptibility experimental results. In addition, the broader electronic states of the *e*_g_ orbitals close to the Fermi level can improve the electron transfer mobility and provide a lower adsorption energy between the active site and reaction intermediate, boosting the eNRR performance.^[^ ^7^, ^43^ ^]^ As illustrated in Figure 7d,e, the broader $d_{x^{2}}-d_{y^{2}}$ electron state enabled more efficient electron transfer between N_2_ and the electrocatalyst, thereby facilitating the N_2_ protonation step. This was verified by examining the projected DOS before and after N_2_ adsorption (Figure 7f). The projected DOS (Figures 7f and S39, Supporting Information) revealed that the Co‐3*d* orbital of Co_9_S_8_/Nb_2_CT_x_‐P and the *N_2_‐2*p* orbital can overlap more effectively compared to those of Co_9_S_8_ (Figure 7f), Co_9_S_8_‐P (Figure 7f), and Co_9_S_8_/Nb_2_CT_x_ (Figure 7f). Therefore, the Co_9_S_8_/Nb_2_CT_x_‐P M–S heterojunction is more conducive to the adsorption and activation of N_2_. Charge difference analysis (Figure S40, Supporting Information) demonstrated that electrons can accumulate at the edge of the interface between Co_9_S_8_ and Nb_2_CT_x_. Both negative (charge accumulation) and positive (charge depletion) charges are simultaneously present around *N_2_ after N_2_ adsorption (Figure 7g).^[^ ^44^ ^]^ Thus, the S_v_s and their neighboring Co return their 3*d* electrons to the π* orbital of *N_2_, further activating and polarizing N_2_ molecules through an “acceptance–donation” mechanism.

We further calculated the Gibbs free energies for the eNRR catalyzed by Co_9_S_8_‐P, Co_9_S_8_/Nb_2_CT_x_‐P, Co_9_S_8_, and Co_9_S_8_/Nb_2_CT_x_ (**Figure** **7h**). Remarkably, the high adsorption ability of N_2_ molecules onto Co_9_S_8_/Nb_2_CT_x_‐P led to a low energy barrier (0.27 eV) for the first protonation RDS of the *N_2_→*NNH formation process. In addition, Co_9_S_8_/Nb_2_CT_x_ and Co_9_S_8_ exhibited the energy barriers of 2.19 and 2.27 eV in the first protonation step, respectively, which were markedly higher than those of Co_9_S_8_/Nb_2_CT_x_‐P (0.27 eV) and Co_9_S_8_‐P (0.34 eV) (Figure 7h). Moreover, the RDS barrier of Co_9_S_8_/Nb_2_CT_x_‐P is lower than those of most previously reported catalysts.^[^ ^45^ ^]^ The competing HER kinetics in the eNRR procedure driven by Co_9_S_8_/Nb_2_CT_x_ and Co_9_S_8_/Nb_2_CT_x_‐P was calculated using DFT, as depicted in Figure S41 (Supporting Information). The result manifested that the Gibbs free energy of hydrogen (*H) adsorption on the Co_9_S_8_/Nb_2_CT_x_‐P (−0.26 eV) was remarkably lower than that of Co_9_S_8_/Nb_2_CT_x_ (0.80 eV). Thereby, the *H species can be spontaneously adsorbed on the Co_9_S_8_/Nb_2_CT_x_‐P surface, finally forming the 1/2H product. These results hinted that the generated rich sulfur vacancies remarkably accelerated the formation of the transition intermediate of active *H species, thus boosting the rate‐determining step of the eNRR, i.e., the production of *NNH. In addition, Co_9_S_8_/Nb_2_CT_x_‐P showed lower energy of N_2_ adsorption (Figure 7c, −0.87 eV) than that of hydrogen adsorption (−0.26 eV). It suggested the efficient HER suppression, thereby improving Faradaic efficiency and NH_3_ yield of Co_9_S_8_/Nb_2_CT_x_‐P. These results indicate that the S_v_ abundance together with the synergistic effect of Co_9_S_8_ with Nb_2_CT_x_ can promote the highly spin‐polarized state of the Co 3*d* orbital electrons. This activates N_2_ and promotes the formation of *NNH, greatly enhancing the eNRR activity.

## 3 Conclusion

In summary, a new Co_9_S_8_/Nb_2_CT_x_‐P hybrid was synthesized by coupling Co_9_S_8_ with Nb_2_CT_x_, in which the spin state of the Co^2+^ catalytic center was successfully regulated to improve the efficiency and selectivity of the eNRR. The transformation of the Co 3*d* orbital electron configuration from low (t_2g_^6^e_g_^1^) to high (t_2g_^5^e_g_^2^) spin was induced by S_v_ and interface effects. The resulting highly spin‐polarized state of the Co_9_S_8_/Nb_2_CT_x_‐P hybrid demonstrated superior eNRR performance in acid and neutral electrolyte. Compared to the pristine Co_9_S_8_ and Co_9_S_8_/Nb_2_CT_x_ hybrids, Co_9_S_8_/Nb_2_CT_x_‐P exhibited high NH_3_ yields of 62.62, and 41.47 µg h^−1^ mg_cat._^−1^ coupled with FEs of 30.33% and 23.19% in 0.1 m HCl and 0.1 m Na_2_SO_4_, respectively, surpassing the performances of most reported NRR electrocatalysts. DFT calculations indicated that this improvement in catalytic performance is related to the increase and upward shift of the Co $d_{x^{2}}-d_{y^{2}}$ orbitals, which significantly promotes the adsorption and activation of N_2_ and reduces its protonation energy barrier. This study demonstrates the potential of heterostructured materials in designing high‐performance spin‐based catalysts, highlighting the importance of optimizing the spin states for the eNRR.

## Conflict of Interest

The authors declare no conflict of interest.

## Supporting information

Supporting Information

## Data Availability

The data that support the findings of this study are available from the corresponding author upon reasonable request.
