# Supplementary material for: Spin Manipulation of Co sites in Co9S8/Nb2CTx Mott–Schottky Heterojunction for Boosting the Electrocatalytic Nitrogen Reduction Reaction
Source: Adv Sci (Weinh). 2024 Sep 3;11(40):2407301. doi: 10.1002/advs.202407301 (PMC11516103; doi:10.1002/advs.202407301)
Supplement: Supplementary file 1 — Supporting Information [file ADVS-11-2407301-s001.pdf]

ADVANCED SCIENCE
Open Access

Supporting Information

for *Adv. Sci.*, DOI 10.1002/advs.202407301

Spin Manipulation of Co sites in Co_9S_8/Nb_2CT_x Mott–Schottky Heterojunction for Boosting the Electrocatalytic Nitrogen Reduction Reaction

Shuai Zhang, Weihua Zhao, Jiameng Liu, Zheng Tao, Yinpeng Zhang, Shuangrun Zhao, Zhihong Zhang and Miao Du**

Supporting Information

Spin manipulation of Co sites in Co_9S_8/Nb_2CT_x Mott-Schottky heterojunction for boosting the electrocatalytic nitrogen reduction reaction

Shuai Zhang, Weihua Zhao, Jiameng Liu, Zheng Tao, Yinpeng Zhang, Shuangrun Zhao, Zhihong Zhang, Miao Du**

S. Zhang, W.H. Zhao, Z. Tao, Y.P. Zhang, S.R. Zhao, Prof. Z.H. Zhang, Prof. M. Du
College of Material and Chemical Engineering, Institute of New Energy Science and Technology, School of Future Hydrogen Energy Technology, Zhengzhou University of Light Industry, Zhengzhou 450001, China

J.M. Liu
School of Medical Engineering, Xinxiang Medical University, Xinxiang 453003, P. R. China
E-mail: 2006025@zzuli.edu.cn; dumiao@zzuli.edu.cn

S1. Experimental section

S1.1 Materials and chemicals

Cobalt acetylacetonate ($C_{15}H_{21}CoO_6$) (99%) and thiourea (CH_4N_2S) were purchased from Aladdin Reagent Co. Ltd. (Shanghai, China). Paradimethylaminobenzaldehyde ($C_9H_{11}N_O$, 99%) was purchased from Shanghai Yuanye Bio-Technology Co., Ltd. Sodium citrate ($Na_3C_6H_5O_7 \cdot 2H_2O$, 99.5%) was obtained from Aladdin. Hydroquinone (99%) and NaOH (96%) were purchased from Tianjin Kermel Chemical Reagent Co., Ltd. Hydrochloric acid (HCl, 36~38%) and acetone (99.5%) were obtained from Luoyang haohua Chemical Reagent Co., Ltd. Ethanol (99%), sodium sulfate (99%), and ammonium chloride (NH_4Cl, 99%) were purchased from Tianjin Damao Chemical Trading Co., Ltd. Sodium nitroprusside ($C_5H_4FeN_6Na_2O_3$, 99%) and salicylic acid ($C_7H_6O_3$, 99.5%) were purchased from Ron Reagent. Hydrazine hydrate (N_2H_4, 5% HCl) was obtained from Guobiao (Beijing) Testing and Certification, Co., Ltd. Ammonia (NH_4OH, 25%) was purchased from Fortune Chemical Reagent Co., Ltd. NaClO (6~14% active chlorine basis) was obtained from Maclin. $^{14}N_2$ gas (99.99%) and Ar gas (99.99%) were obtained from Huanyu Co., Ltd. $^{15}N_2$ gas (99.99%) was obtained from Tianjin Taiya Co., Ltd. The aqueous solutions were prepared with deionized water and all the chemicals were of analytical grade and used without further purification.

S1.2 Preparation of the Co_9S_8/Nb_2CT_x M-S heterojunction before and after the solution plasma (SP) modification

The Co_9S_8/Nb_2CT_x Mott-Schottky (M-S) heterojunction was prepared via the

hydrothermal method. Typically, 249 mg of cobalt acetylacetonate ($C_{15}H_{21}CoO_6$) and 76 mg of thiourea (CH_4N_2S) were simultaneously dispersed in the 10 mL of the Nb_2CT_x MXene suspension (3 mg mL^{-1}), following by ultrasonic treatment for 30 min. After fully mixed, the resulting solution was transferred into a Teflon-lined stainless-steel autoclave (100 mL) and then kept at 200 °C for 48 h. The prepared Co_9S_8/Nb_2CT_x-1 heterojunction was separately washed with water and ethanol thrice, and dried in a vacuum oven at 60 °C. To investigate the effect of Co_9S_8 content on the eNRR performance of Co_9S_8/Nb_2CT_x heterojunctions, varying dosages of cobalt acetylacetonate (125 mg and 498 mg) and thiourea (38 mg and 152 mg) were employed in the preparation of a series of Co_9S_8/Nb_2CT_x heterojunctions using the same method with Co_9S_8/Nb_2CT_x-1. The resulting heterojunctions were defined as Co_9S_8/Nb_2CT_x-2 and Co_9S_8/Nb_2CT_x-3, respectively. Additionally, Co_9S_8 was synthesized under similar conditions without the addition of Nb_2CT_x for comparison purposes.

The Co_9S_8/Nb_2CT_x-P M-S heterojunction was prepared via the SP modification method. Typically, 2 mg Co_9S_8/Nb_2CT_x was dispersed in 1.9 mL of water in a glass vial. Then, the glass vial was stood on the platform in the plasma chamber and modified by the SP method in an HQ-2 plasma enhanced chemical vapor deposition system (the radio frequency of 13.56 MHz, the input power of 200 W for 10 min, continuous wave, and a constant pressure of 0.1 Pa), which was manufactured by the Institute of Microelectronics of the Chinese Academy of Sciences. Subsequently, the suspension was filtered and dried in air, following by thoroughly rinsing with Milli-Q water thrice. After dried in air, the resultant powder was then denoted as Co_9S_8/Nb_2CT_x-P. For

comparison, Nb_2CT_x and Co_9S_8 were treated using the similar way, named by the Nb_2CT_x-P and Co_9S_8-P, separately.

S1.3 Electrochemical nitrogen reduction reaction (NRR) measurements

Electrochemical measurements were performed on an H-type-cell equipped with a CHI 760E workstation (CH Instruments, Inc., Shanghai, China), which was separated by the Nafion 117 membrane. The Co_9S_8/Nb_2CT_x-P M-S heterojunction was used as the working electrode, while Ag/AgCl (saturated KCl solution) and graphite rod worked as the reference and counter electrodes, respectively. The electrolyte was composed of 0.1 M HCl water (0.1 M Na_2SO_4) solution. Before each the eNRR process, the electrochemical system was first bubbled continuously with N_2 gas (Ar gas) for at least 30 min. Then, the gas was maintained throughout electrochemical reaction. Additionally, the nitrogen gas outlet was positioned close to the surface of the electrocatalyst-coated electrode, thus creating a three-phase gas-solid-liquid configuration. As a result, the low solubility and slow gas diffusion barriers can be overcome because of the direct N_2 supply pathway to the electrocatalyst surface cyclic voltammetry (CV) tests were carried out at a scan rate of 5 mV s^{-1} between the applied potential of –0.6 V to –0.2 V (-0.6 V to -0.3 V, -0.8 V to -0.4 V) vs reversible hydrogen electrode (RHE) in N_2-saturated electrolyte. Potentiostatic measurements were carried out at a series of applied potentials including –0.2 V, –0.3 V, –0.4 V, –0.5 V, and -0.6 V vs RHE for 6000 s under constant room temperature. The potentials reported in this work were converted to the RHE scale via the calibration with the following equation: E (*vs.* RHE) = E (*vs.* Ag/AgCl) + 0.0592×pH.

S1.4 Construction of $Zn-N_2$ battery

Base on the excellent eNRR performance of Co_9S_8/Nb_2CT_x-P, an $Zn-N_2$ battery was constructed with 5 mg of Co_9S_8/Nb_2CT_x-P coated with $1\times0.5\ cm^2$ carbon paper (CP) as the cathode and zinc foil as the anode. The constant current charging-discharging performance measurements were carried out by a CHI760E workstation instrument at room temperature. The electrochemical reactions on each electrode in acidic and neutral electrolyte are described as follows:

Cathode reaction: $N_2 + 6H_2O + 6e^- \rightarrow 2NH_3 + 6OH^-$ (neutral electrolyte) (1)

$N_2 + 6H^+ + 6e^- \rightarrow 2NH_3$ (acidic electrolyte) (2)

Anode reaction: $Zn \rightarrow Zn^{2+} + 2e^-$ (acidic and neutral electrolyte) (3)

Overall reaction: $N_2 + 6H_2O + 3Zn \rightarrow 2NH_3 + 6OH^- + 3Zn^{2+}$ (neutral electrolyte) (4)

$3Zn + N_2 + 6H^+ \rightarrow 3Zn^{2+} + 2NH_3$ (acidic electrolyte) (5)

S1.5 Basic characterizations

Field emission scanning electron microscopy (SEM) images were obtained with a JSM-6700F (JEOL) operating at 5 kV in LABE mode. Transmission Electron Microscop (TEM) images were obtained with a Tecnai F20 electron microscope operated at 80 kV. Atomic force microscope (AFM) images of the samples were analyzed by a Bruker Dimension Icon AFM. X-ray photoelectron spectroscopy (XPS) was taken to analyze the chemical compositions and bond characters by using PHI Quantera SXM with monochromatic Mg X-ray radiation source. X-ray diffraction (XRD) patterns were recorded on a Bruker GADDS XRD diffractometer with Cu KI radiation to obtain the structures of composites. Composition and concentration

analyses of the nanoparticle suspension were conducted using a Thermo Scientific iCAP 6500 model ICP-OES. Nitrogen absorption-desorption tests were performed on a Micromeritics ASAP 2020 adsorption apparatus at 77 K up to 1 bar. The porosity including specific Langmuir and Brunauer-Emmett-Teller (BET) surface areas, pore volume, and pore size was obtained by analyzing nitrogen adsorption-desorption isotherms with Micromeritics ASAP 2020 built-in software. ^{1}H NMR spectra were recorded on a Bruker NMR spectrometer (400 MHz) by using DMSO-d6 as solvent. X-ray absorption spectroscopy (XAS) was conducted in transmission mode at beamline station 4B9A of the Beijing Synchrotron Radiation Facility (BSRF). Data reduction, analysis, and EXAFS fitting were carried out using the Athena and Artemis programs from the Demeter data analysis packages, which employ the FEFF6 program for fitting the EXAFS data. The electron paramagnetic resonance (EPR) spectra were carried out on a Bruker EMX PLUS spectrometer operating at the X band with a continuous wave (CW) setup. The spectrometer was equipped with a superhigh Q (ER 4122 SHQ) resonator. EPR spectra were acquired at 298 K using a microwave frequency of 9.83 GHz, 15.0 dB microwave attenuation, 3.0 G modulation amplitude, and 100 kHz modulation frequency. The temperature dependent magnetizations (*M*) measurements were carried out under the magnetic-filed strength (*H*) of 1 kOe for all samples. The effective magnetic moments (μ_{eff}) of all samples were obtained according to $\mu_{eff} = \sqrt{8C}\mu_B$, where μ_B is Bohr magneton and C is Curie constant. In high temperature domain (above 150 K), the susceptibility derived from the magnetizations (χ=M/H) obeys a paramagnetic Curie–Weiss law: χ=C/(T-T_c), where T_c is Curire-Weiss

temperature.

S1.6 Detection of ammonia (NH_3)

Indophenol blue method was adopted to estimate the concentration of NH_3 in 0.1 M HCl or 0.1 M Na_2SO_4 electrolyte after electrolysis for 6000 s. The color reagent system was prepared as follows: Solution A consisted of a 1 M NaOH solution containing 5 wt% salicylic acid and 5 wt% sodium citrate; Solution B was a 0.05 M NaClO solution; and Solution C was a 1 wt% sodium nitroferricyanide ($C_5FeN_6Na_2O$) aqueous solution. The concentration-absorbance curves were established using a series of the standard NH_3 solutions. First, the post-tested electrolyte solution (2.0 mL) was removed from electrochemical system, followed by the sequential addition of 2.0 mL solution A and 1 mL solution B and 0.2 mL solution C. After keeping for 2 h at room temperature, the absorption spectrum was measured on an ultraviolet-visible (UV-vis) spectrophotometer. The absorbance intensity at 655 nm was utilized to estimate the yield of NH_3 based on the standard curve.

S1.7 Detection of hydrazine (N_2H_4)

The concentration of N_2H_4 was spectrophotometrically determined using Watt and Chrisp method. A mixture of para-(dimethyl amino) benzaldehyde (5.99 g), HCl (concentrated, 30 mL), and ethanol (300 mL) was used as a color reagent. Actually, 3 mL of the solution was taken out from the electrochemical reaction vessel, following by adding the above color reagent. The mixture was kept stirring for 10 min at room temperature. The amount of N_2H_4 formed during the electrolysis was determined based on the absorbance intensity at 455 nm. The concentration-absorbance curve was

established by the standard N_2H_4 solution, which contains the same concentration of HCl as used in each electrolysis experiment.

S1.8 Calculation of the NH_3 yield rate and Faradaic efficiency (FE)

The FE for eNRR was defined as the ratio of electric charge used for synthesizing NH_3 divided by the total charge passed through the electrodes during electrolysis.

$$Y = 0.4002X + 0.06145$$

$$r(NH_3) = 3600 \times \frac{X \times 30}{6000}$$

Among them, Y is the value obtained by UV-vis (Ultraviolet-visible Spectrophotometer) testing the solution after color development, and r(NH_3) is the rate of the NH_3 production.

Assuming three electrons were required to produce one NH_3 molecule, the Faradaic efficiency could be calculated as:

$$FE(NH_3) = \frac{3 \times \mathrm{F} \times \frac{[NH_3]}{17} \times V}{1000000 \times \int idt}$$

where [NH_3] is the measured concentration of NH_4^+, V is the volume of HCl electrolyte in the cathode chamber, *i* is the instantaneous current measured by chronoamperometry.

S1.9 Isotope labeling experiment

Briefly, the labeled $^{15}N_2$ as the feed gas was purified by passing it through the NaOH solution, $KMnO_4$ solution, and then a volumetric flask containing the two-thirds Na_2SO_4 solution to remove any nitrogen contamination. In the electrocatalytic N_2 reduction experiment, Ar gas was purged into the cathodic cell to remove impurity and then purged for 30 min with the test gas. After electrolysis in 0.1 M HCl solution for 6000 s, 20 mL of the electrolyte was concentrated to 2 mL by heating via reduced

pressure distillation. Subsequently, 0.9 mL of the resulting solution was mixed with 0.1 mL DMSO-d6 for the ^{1}H-NMR measurement. The ^{1}H NMR spectroscopy was performed obtained using this method.

S2. Basic characterizations

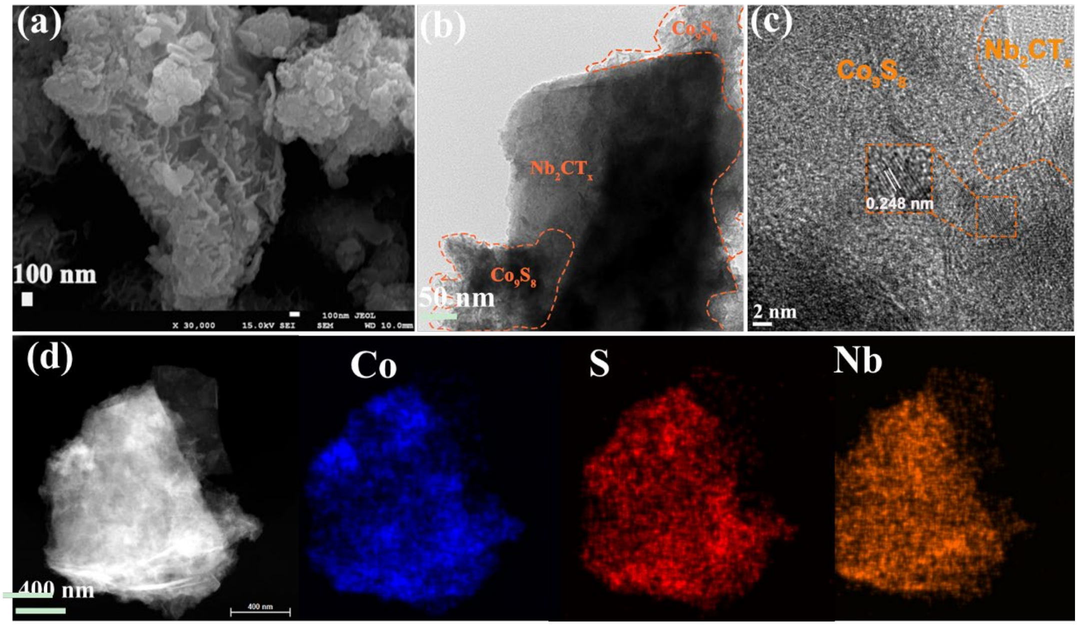

Figure S1 (a) High-magnification FE-SEM, (b, c) TEM and high-resolution TEM (HR-TEM), and (d) EDS mapping images of Co_9S_8/Nb_2CT_x.

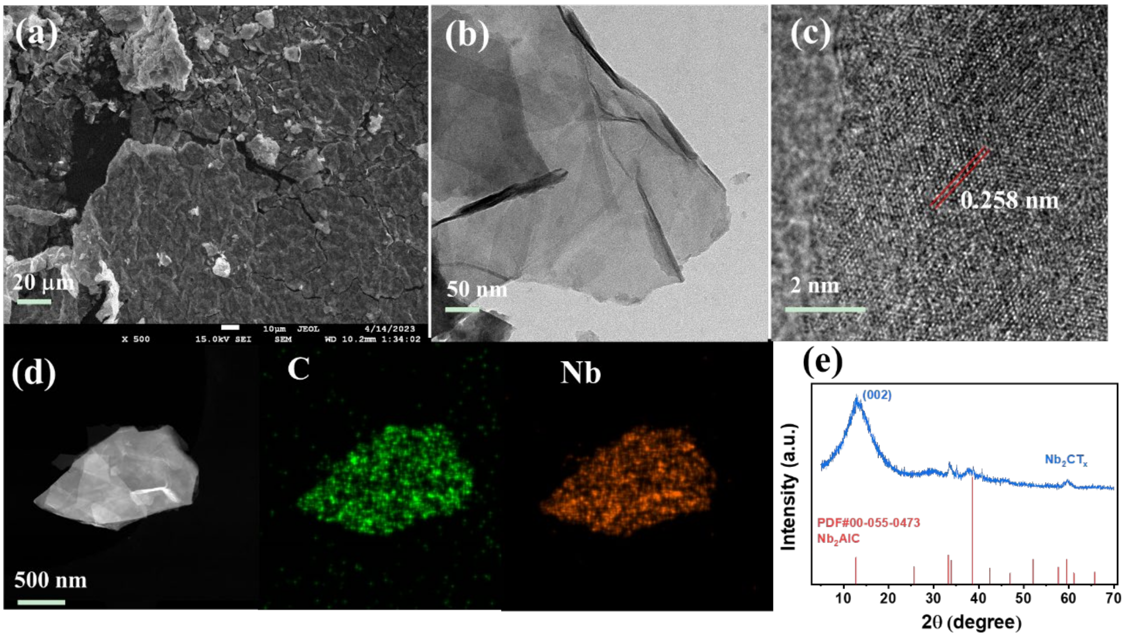

Figure S2 (a) High-magnification FE-SEM image, (b, c) TEM and HR-TEM images, and (d) EDS mapping spectrum of Nb_2CT_x MXene. (e) XRD pattern of Nb_2CT_x.

Discussion: The SEM image of Nb_2CT_x (**Figure S2a**) indicates an accordion-like structure, for which the few layers nanostructure of Nb_2CT_x hints the full removal of Al layers from the MAX phase[1]. The few layers feature of Nb_2CT_x is further proved by the TEM image of Nb_2C MXene (Figure **S2b**), in which the exfoliated Nb_2CT_x nanosheets are stacked together. The high-resolution TEM (HR-TEM) image (Figure **S2c**) shows a clear lattice spacings of 0.258 nm, attributing to the (111) plane of Nb_2CT_x. In addition, the hexagonal symmetry structure was also verified by the selected area electron diffraction (SAED) pattern (Figure **S2d**). The chemical composition of the Nb_2CT_x was investigated by energy-dispersive X-ray spectroscopy (EDS), where the Nb and C elements originating from the interior of the material and the O element can be assigned to the surface terminations of the Nb_2CT_x flakes (Figure **S2d**). The

diffraction peak at 2θ = 38.9° due to the (103) plane of Nb_2AlC (PDF#00-055-0473) disappears after etching, indicating the successful preparation of Nb_2CT_x.

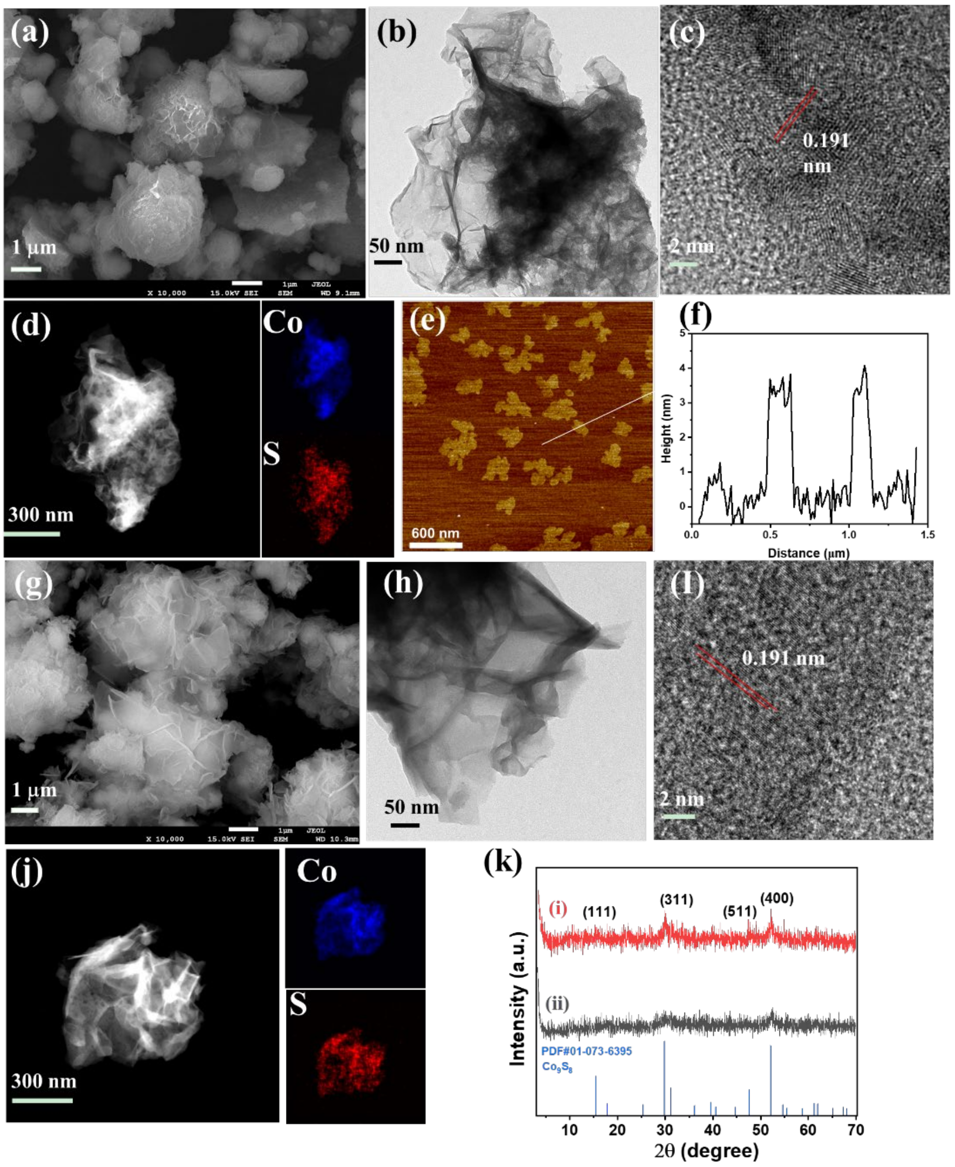

Figure S3 (a) High-magnification FE-SEM image, (b, c) TEM and HR-TEM images, and (d) EDS mapping spectrum of Co_9S_8. (e) AFM image of Co_9S_8 and (f) corresponding specific height profile. (g) High-magnification FE-SEM image, (h, I) TEM and HR-TEM images, and (j) EDS mapping spectrum of Co_9S_8-P. (k) XRD patterns of (i) Co_9S_8 and (ii) Co_9S_8-P.

Discussion: The FE-SEM images of the samples before and after the SP treatment (Figures **S3a** and **g**) are very similar, indicating a nanoflower structure composed of

many ultra-thin nanosheets. It further confirmed by the TEM images of Co_9S_8 (Figure **S3b**) and Co_9S_8-P (Figure **S3h**). The HR-TEM image (Figures **S3c** and **S3I**) illustrates a clearly lattice spacing of 0.191 nm, corresponding to the (111) plane of the Co_9S_8 nanosheets. As shown in Figures **S3**e and **f**, atomic force microscopy (AFM) image and the corresponding height profile of Co_9S_8 reveal a 2D nanosheet morphology with a thickness of approximately 3.5 nm. The EDS mapping images of Co_9S_8 before and after the SP modification (Figures **S3d** and **S3j**) illustrate that Co and S elements are uniformly distributed the selected region. The diffraction peaks at 2θ = 15.3°, 29.9°, 47.4°, and 52.4°, corresponding to the (111), (311), (511), and (400) crystal faces of Co_9S_8 (PDF#01-073-6395), respectively (Figure **S3k**). No significant change is observed in the XRD pattern of Co_9S_8 before and after the SP treatment.

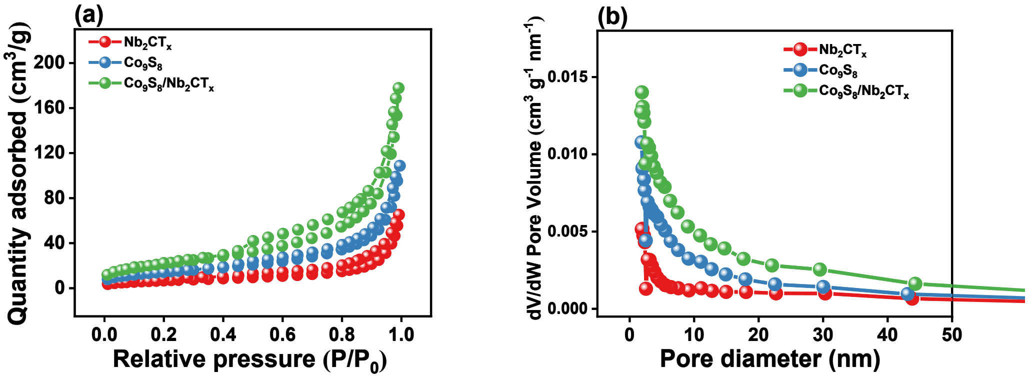

Figure S4 (a) Nitrogen adsorption-desorption isotherm and (b) HK pore size patterns of (i) Nb_2CT_x, (ii) Co_9S_8, and (iii) Co_9S_8/Nb_2CT_x.

Table S1 Summary of surface areas, pore volumes and mean pore sizes of Co_9S_8, Nb_2CT_x, and Co_9S_8/Nb_2CT_x.

**Catalysts**	**BET surface area (m^2 g^{-1})**	**V (cm^3 g^{-1})**	**mean pore size (Å)**
Nb_2CT_x	24.724	0.010	7.863
Co_9S_8	51.8890	0.021	7.825
Co_9S_8/Nb_2CT_x	80.1403	0.032	7.726

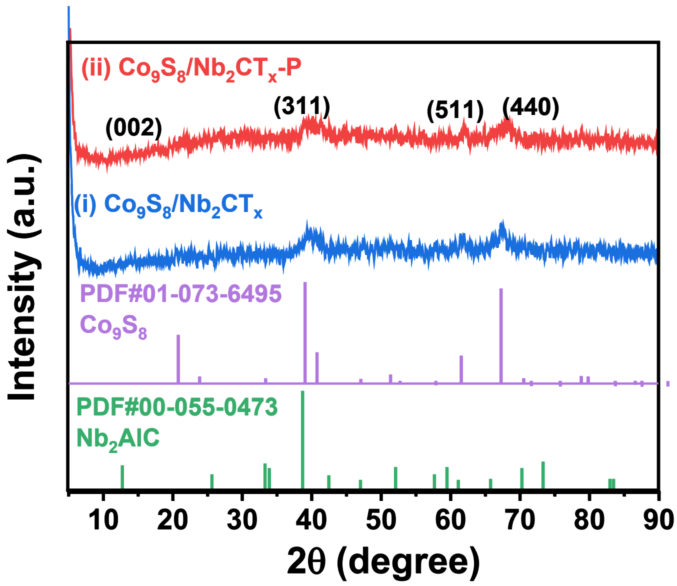

Figure S5 XRD patterns of (i) Co_9S_8/Nb_2CT_x, and (ii) Co_9S_8/Nb_2CT_x-P.

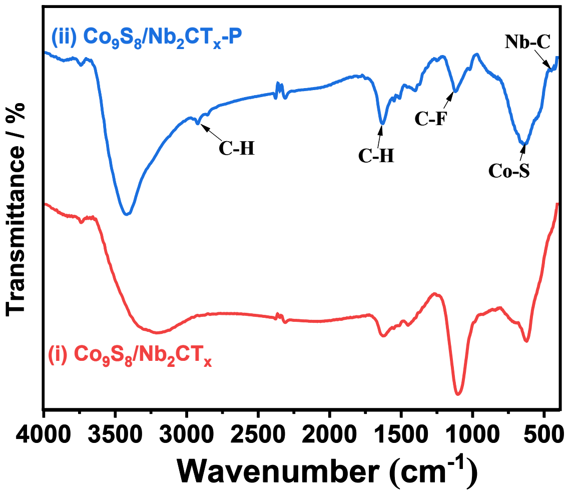

Figure S6 FT-IR spectra of (i) Co_9S_8/Nb_2CT_x, and (ii) Co_9S_8/Nb_2CT_x-P.

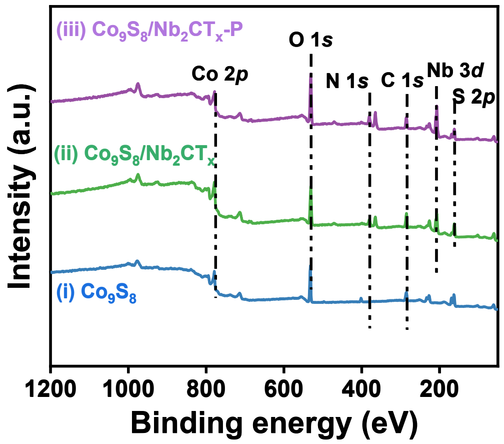

Figure S7 XPS survey scan spectra of (i) Co_9S_8, (ii) Co_9S_8/Nb_2CT_x, and (iii) Co_9S_8/Nb_2CT_x-P.

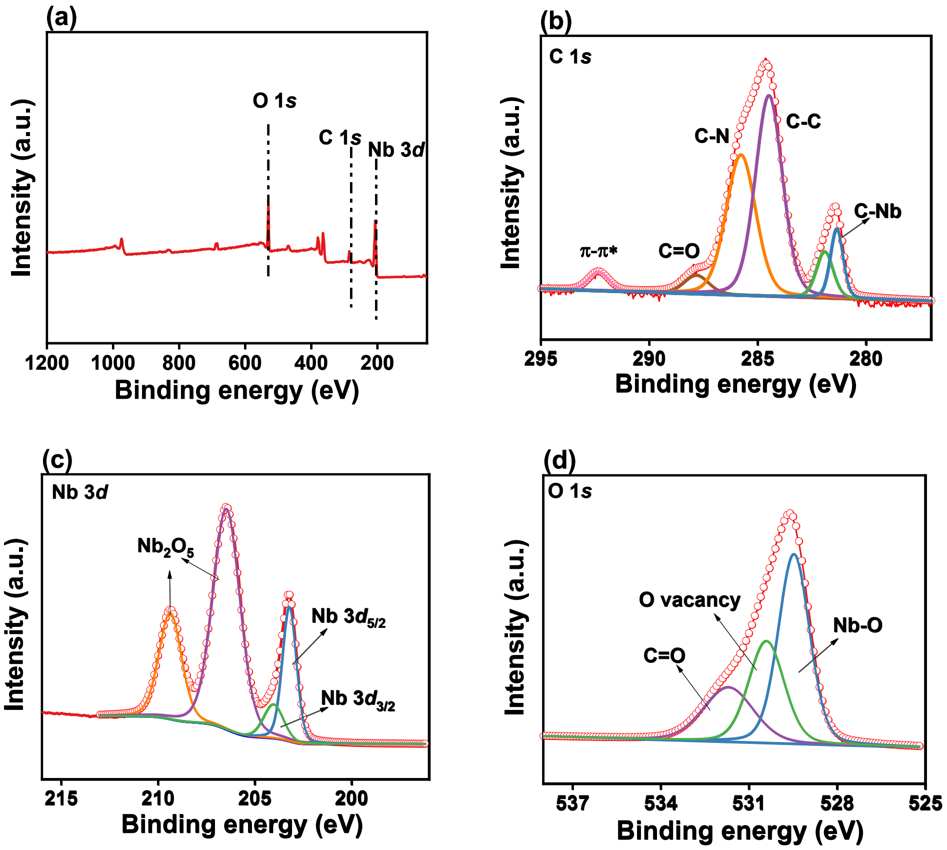

Figure S8 (a) XPS survey scan spectra of Nb_2CT_x. High-resolution (b) C 1*s*, (c) Nb 3*d*, and (d) O 1*s* XPS spectra of Nb_2CT_x.

Discussion: The XPS survey scan spectrum of the Nb_2CT_x (**Figure S8a**) shows the clear signals of Nb 3*d* (207.3 eV) C 1*s* (284.1 eV), and O 1*s* (531.6 eV). The results show that Nb is partially oxidized during the preparation of Nb_2CT_x. The C 1*s* XPS spectrum (**Figure S8a**) can be divided into the main components of C-C (284.5 eV) and C-N (285.8 eV), accompanying with C=O (287.7 eV) and π–π^* (292.34 eV). The two additional peaks at the binding energies (BEs) of 281.4 and 281.9 eV are due to C-Nb bonding, which are consistent with the analysis of Nd 3*d* XPS spectrum. **Figure S8b** shows that the high-resolution Nb 3*d* XPS spectrum can be deconvoluted into two small peaks at the BEs of 203.4 and 204 eV, due to Nb-C 3$d_{5/2}$ and Nb-C 3$d_{3/2}$, respectively,

along with the strong peaks at BEs of 206.5 and 209.4 eV attributed to Nb_2O_5 3$d_{5/2}$ and Nb_2O_5 3$d_{3/2}$, respectively [2-3]. As for the O 1s XPS spectrum (**Figure S8c**), the part of Nb-O (529.5 eV) is obtained, which in line with the peak of Nb 3*d* peak, along with oxygen vacancy (530.4 eV) and C=O (531.7 eV).

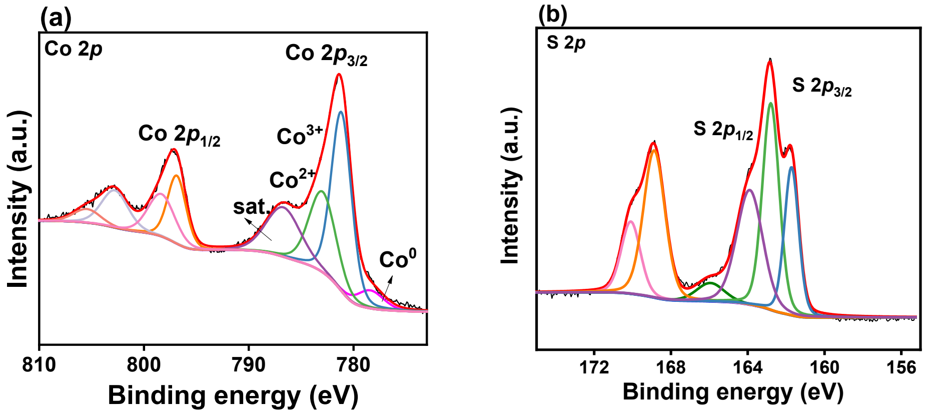

Figure S9 High-resolution (a) Co 2*p* and (b) S 2*p* XPS spectra of Co_9S_8.

Discussion: As for the Co 2*p* XPS spectrum (**Figure S9a**), two clear couples of peak groups can be observed at the BE centers of 778.5 and 793.6 eV, which are due to Co $2p_{3/2}$ and Co $2p_{1/2}$ species, respectively. The Co $2p_{3/2}$ species can be deconvoluted into five components, including 778.5, 779.2, 780.9, 783, and 785.8 eV, which are due to Co^0, Co^{3+}, Co^{2+}, and their satellite shakeup peaks, respectively. The same deconvolutions are observed in the Co $2p_{1/2}$ part. These results suggest the Co species is composed the mixed states, in which the partial Co ions were reduced to the metallic state (Co^0). The existence of Co^0 can remarkably improve the electrochemical activity and catalytic performance of Co_9S_8. The S 2*p* XPS peak at 161.7 and 162.8 eV are ascribed to the S $2p_{3/2}$ and S $2p_{1/2}$ orbitals of Co_9S_8 (**Figure S9b**).

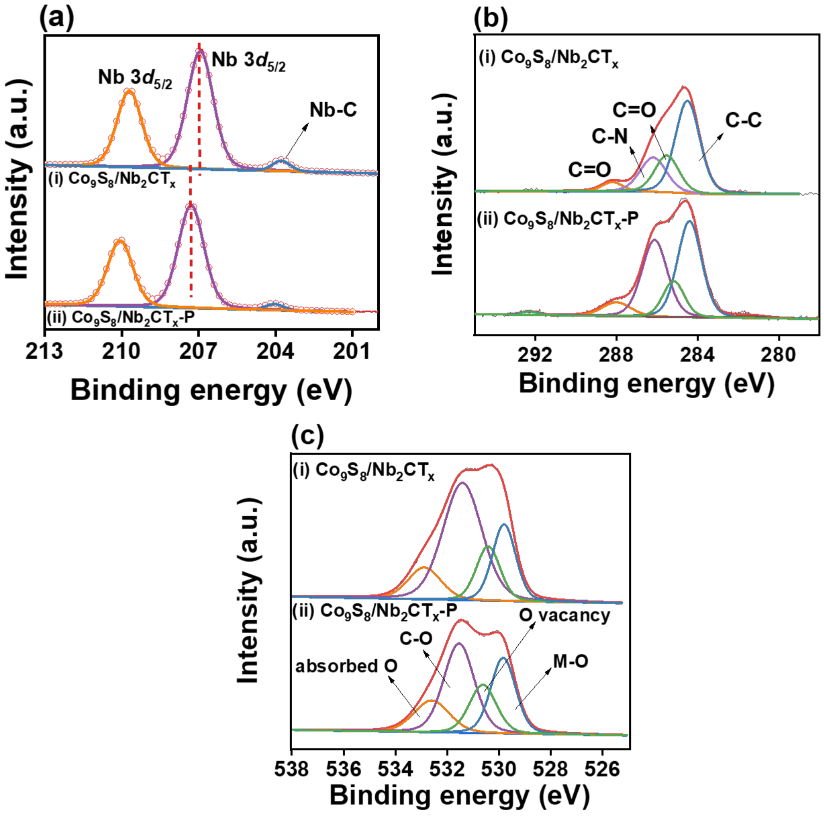

Figure S10 High-resolution (a) Nb 3*d*, (b) C 1*s* and (c) O 1*s* XPS spectra of (i) Co_9S_8/Nb_2CT_x, and (ii) Co_9S_8/Nb_2CT_x-P.

Discussion: The Nd 3*d* XPS spectrum of Co_9S_8/Nb_2CT_x-P (curve *i*, **Figure S10a**) can be divided into Nb-C (204.0 eV), Nb-O $3d_{5/2}$ (207.3 eV) and Nb-O $3d_{3/2}$ (210.1 eV), showing the positive shifts by 0.9, 0.8, and 0.7 eV in contrast to Co_9S_8/Nb_2CT_x, respectively. This is caused by the formation of S defect after plasma treatment. In addition, the peak position of Nd $3d_{5/2}$ and Nd $3d_{3/2}$ of Co_9S_8/Nb_2CT_x (curve *ii*, **Figure S10a**) shift negatively by 0.42 eV and 0.38 eV compare to those of Nb_2CT_x. This result further reveals the electron transfer occurs from Nb_2CT_x to Co_9S_8. These results can be further confirmed by the ultraviolet photoelectron spectroscopy (UPS) analysis. In addition, the C 1*s* XPS (curve *ii*, **Figure S10b)** peak of Co_9S_8/Nb_2CT_x-P comprises C-

C (284.5 eV), C-N (285.2 eV), C-O (286.2 eV), COO (288.2 eV), along with weak peaks of Nb-C (282 eV) and π−π stacking (292.4 eV). As compared, the content of Nb-C species in Co_9S_8/Nb_2CT_x-P is significantly lower than that of Co_9S_8/Nb_2CT_x (curve *i*, **Figure S10b**). The XPS splitting of the O 1*s* peak of Co_9S_8/Nb_2CT_x-P (curve *ii*, **Figure S10c)** comprises Nb-O (529.8 eV), oxygen vacancy (530.4 eV), and C=O (531.3 eV), and C-O (532.5 eV), resemble with those of Co_9S_8/Nb_2CT_x (curve *i*, **Figure S10c)**.

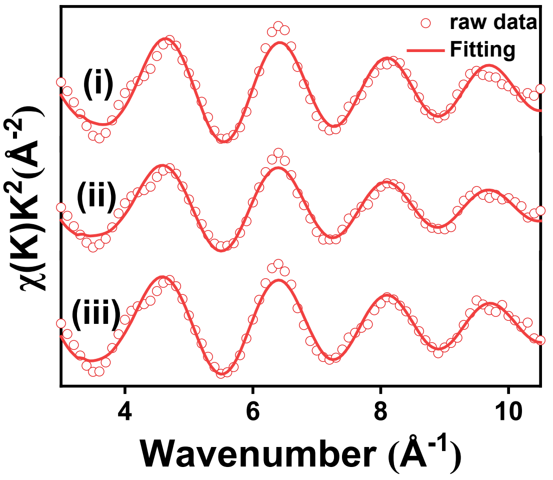

Figure S11 The corresponding K-edge Fourier-transform EXAFS of (i) Co_9S_8, (ii) Co_9S_8/Nb_2CT_x, and (iii) Co_9S_8/Nb_2CT_x-P.

Table S2 Coordination number of Co extracted from the EXAFS fitting.

Samples	Path	N	ΔE (eV)	100R (Å)	$1000\sigma^2$ ($Å^2$)	R-factor
Co foil	Co-Co	12*	6.47	2.49	0.0065	0.018
CoO	Co-N	4.0±0.7	0.22	2.12	0.0074	0.003
Co_9S_8	Co-S1	1.53	-2.70	3.01	0.0036	0.00138
	Co-S2	3.19	14.50	2.09	0.0034	
Co_9S_8/Nb_2CT_x	Co-S1	1.47	-0.92	2.22	0.0029	0.0088
	Co-S2	3.32	14.50	2.10	0.0033	
Co_9S_8/Nb_2CT_x-P	Co-S1	1.26	-1.83	2.22	0.0036	0.0072
	Co-S2	3.11	14.50	2.08	0.0034	

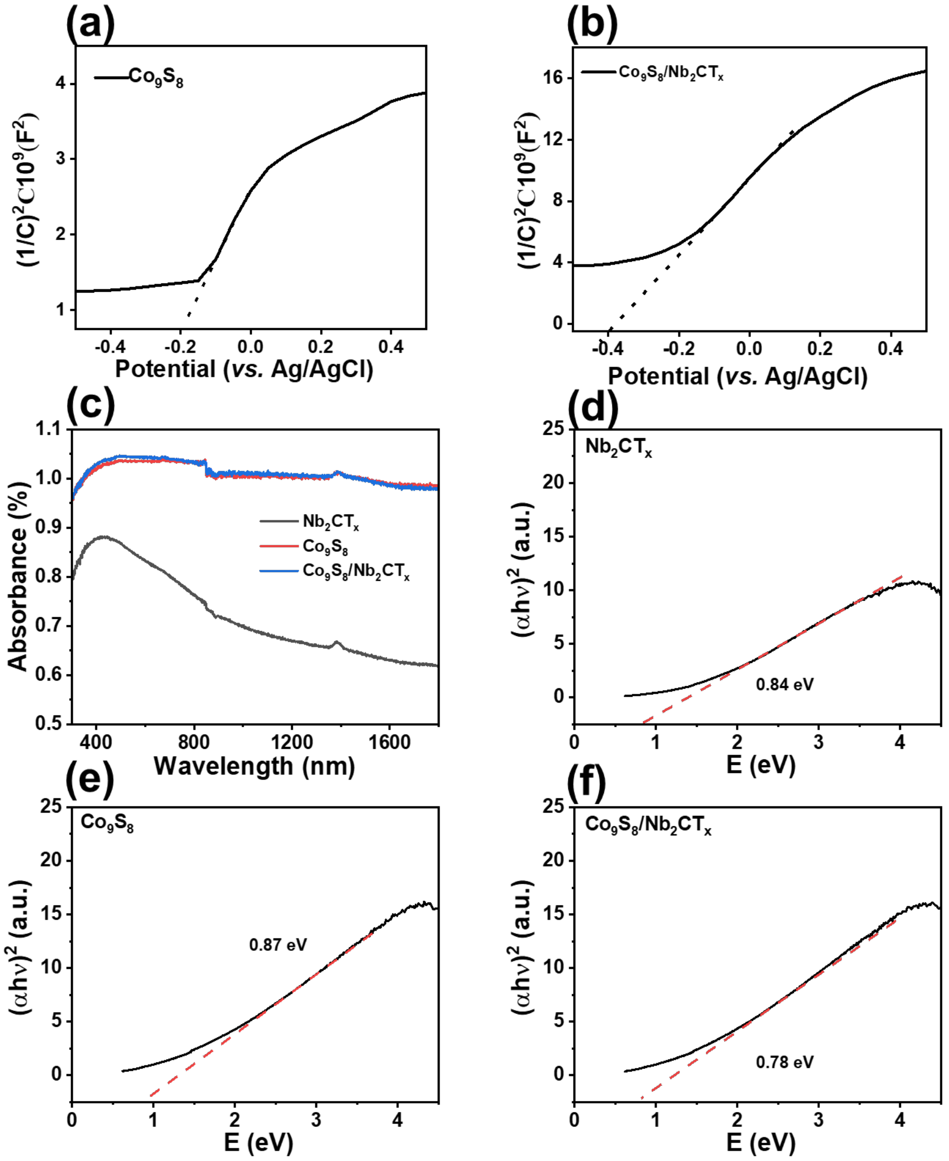

Figure S12 Mott-Schottky curves of (a) Co_9S_8 and (b) Co_9S_8/Nb_2CT_x catalysts in 0.5 M Na_2SO_4 at −1.0 to 0.0 V versus Ag/AgCl. (c) UV-vis diffusion reflectance spectra of Nb_2CT_x, Co_9S_8, and Co_9S_8/Nb_2CT_x. The corresponding Tauc plots of (d) Nb_2CT_x, (e) Co_9S_8, and (f) Co_9S_8/Nb_2CT_x

Discussion: The Fermi level (E_F) of one electrocatalyst is determined by work function measured by UPS, which was performed by using HeI excitation source of 21.22 eV. The work function of one electrocatalyst was deduced by extrapolating the linear section of the UPS to the baseline according to the equation: $\Phi = h\gamma - (E_{cutoff} - E_F)$,

where $h\gamma$ is 21.22 eV, E_{cutoff} is low-energy cutoff edge and E_F is the Fermi level. The MS analysis hints that Co_9S_8 (**Figure S12a**) and Co_9S_8/Nb_2CT_x (**Figure S12b**) show the positive slopes, corresponding to *n*-type semiconductors. Furthermore, the flat-band potential (E_{fb}) can be calculated by MS analysis. The E_{fb} of Co_9S_8 and Co_9S_8/Nb_2CT_x are 0.005 and 0.203 V versus NHE. Additionally, the conduction potential (E_{CB}) of N-type semiconductors is lower than E_{fb} (approximating to 0.2 V). Therefore, the E_{CB} of Co_9S_8 is 0.195 V versus NHE. Figure **S12c** presents UV-vis diffusion reflectance spectra of Nb_2CT_x, Co_9S_8, and Co_9S_8/Nb_2CT_x. The band gap (E_g) can be obtained by using Tauc plots derived from UV-vis spectra based on Kubelka-Munk function (Figures **S12d-f**). Therefore, the E_g of Nb_2CT_x, Co_9S_8, and Co_9S_8/Nb_2CT_x, are 0.84, 0.87. and 0.78 eV, respectively. The valence band potential (E_{VB}) of Co_9S_8 is calculated to 0.675 V based on the equation: $E_{VB}=E_g+E_{CB}$.

S3. The eNRR performance of the developed electrocatalysts

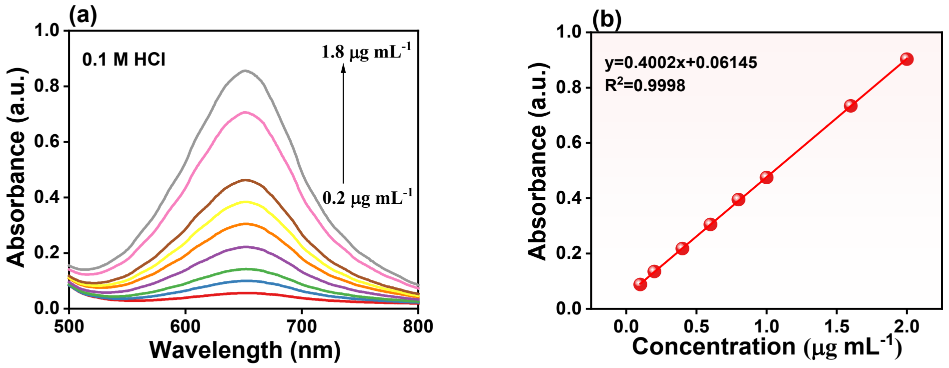

Figure S13 Calibration of the indophenol blue method for subsequent ammonia quantification. (a) Absorbance spectra of indophenol blue in NH^{4+} solutions at various concentrations. (b) Linear correlation of the absorbance intensity to NH^{4+} concentration (inset shows the formation of indophenol blue).

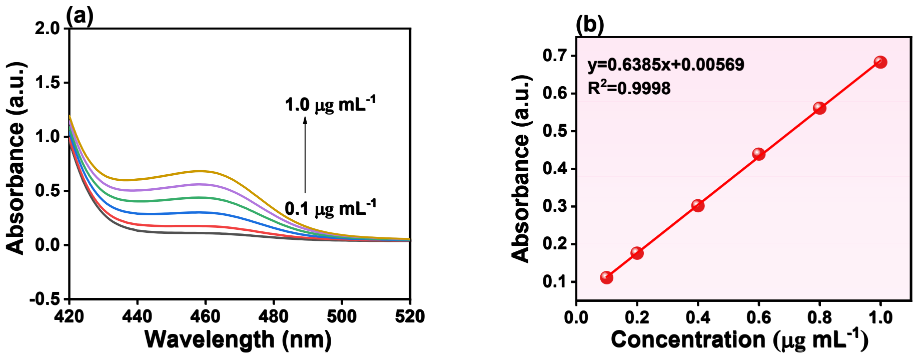

Figure S14 Calibration of the estimation method for subsequent hydrazine quantification. (a) Absorbance spectra of N_2H_4 solutions with various concentrations after incubated for 20 min at room temperature. (b) Calibration curve used for calculation of N_2H_4 concentration.

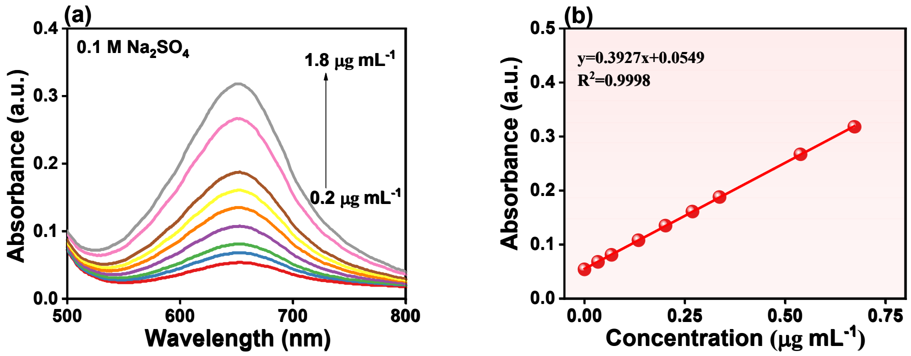

Figure S15 Calibration of the indophenol blue method for subsequent ammonia quantification. (a) Absorbance spectra of indophenol blue in NH^{4+} solutions at various concentrations. (b) Linear correlation of the absorbance intensity to NH^{4+} concentration.

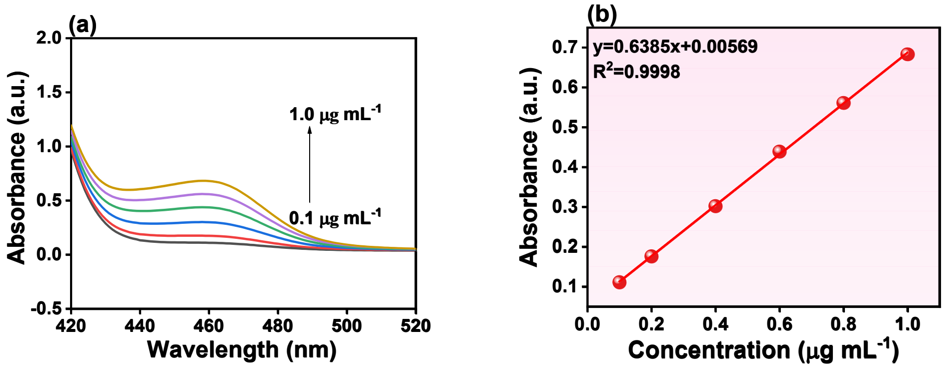

Figure S16 Calibration of the estimation method for subsequent hydrazine quantification. (a) Absorbance spectra of N_2H_4 solutions with various concentrations after incubated for 20 min at room temperature. (b) Calibration curve used for calculation of N_2H_4 concentration.

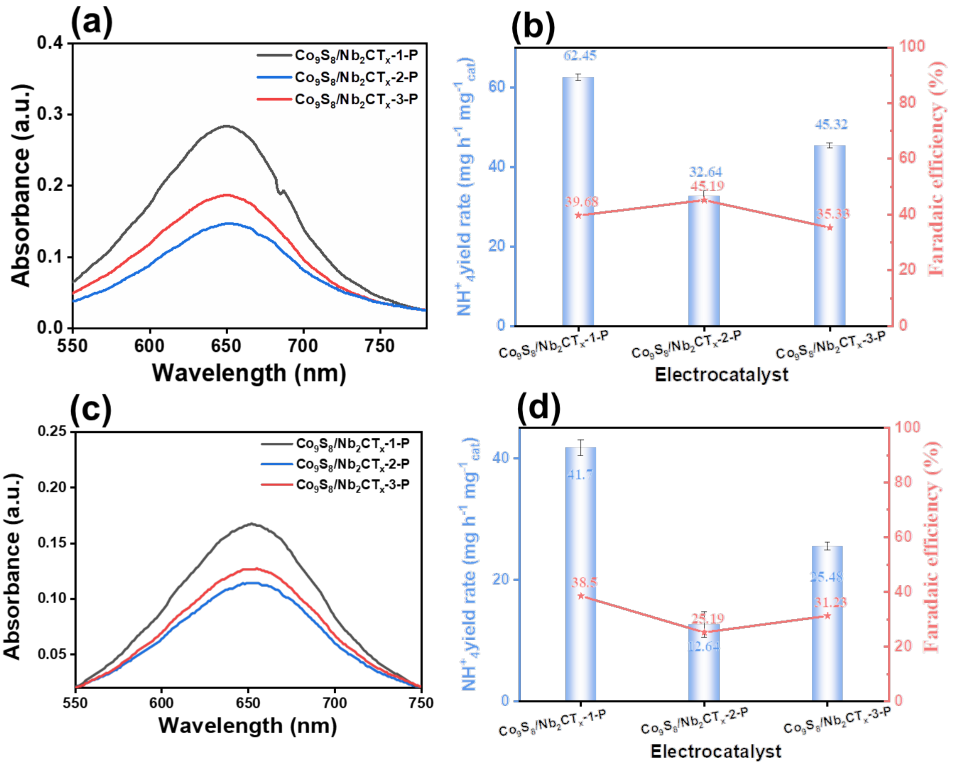

Figure S17 (a) UV-vis absorption spectra of different Co_9S_8 mass contents in Co_9S_8/Nb_2CT_x-P in 0.1 M HCl electrolyte stained with indophenol indicator after electrolysis at applied potential of -0.4 V versus RHE for 6000 s. (b) Dependence of ammonia yield and FE on different Co_9S_8 mass contents in Co_9S_8/Nb_2CT_x-P at an applied potential of -0.4 V versus RHE in N_2-saturated 0.1 M HCl with an NRR measurement time of 6000 s. (c) UV-vis absorption spectra of different Co_9S_8 mass contents in Co_9S_8/Nb_2CT_x-P in 0.1 M Na_2SO_4 electrolyte stained with indophenol indicator after electrolysis at applied potential of -0.6 V versus RHE for 6000 s. (b) Dependence of ammonia yield and FE on different Co_9S_8 mass contents in Co_9S_8/Nb_2CT_x-P at an applied potential of -0.6 V versus RHE in N_2-saturated 0.1 M Na_2SO_4 with an NRR measurement time of 6000 s.

Discussion: In prior to the systematically research on the eNRR ability, th the dosage of cobalt acetylacetonate (125 mg, 249 mg, and 498 mg) and thiourea (38 mg, 76 mg,

and 152 mg) was altered for the preparation of the series of Co_9S_8/Nb_2CT_x heterojunctions contained different contents of Co_9S_8 to optimize the eNRR performance. The obtained heterojunctions were defined by Co_9S_8/Nb_2CT_x-1, Co_9S_8/Nb_2CT_x-2, and Co_9S_8/Nb_2CT_x-3, separately. Correspondingly, the prepared heterojunctions were modified by the solution plasma modification method, obtaining the Co_9S_8/Nb_2CT_x-P electrocatalysts contained diverse Co_9S_8 contents. Further, the influence of the Co_9S_8 content in Co_9S_8/Nb_2CT_x-P on the NRR performances in 0.1 M HCl and 0.1 M Na_2SO_4 was investigated. **Figure S17** demonstrated that the NH_3 yields and FEs derived from the chronoamperometry at the optimized potentials of -0.4 V versus RHE in 0.1 M HCl and -0.6 V versus RHE in 0.1 M Na_2SO_4. Among the three kinds of electrocatalysts, the Co_9S_8/Nb_2CT_x-2 treated by the plasma modification, referred to as Co_9S_8/Nb_2CT_x-P in this work, showed the best eNRR performance with the highest ammonia yields and FEs in both acidic and neutral electrolytes. Therefore, the Co_9S_8/Nb_2CT_x-P electrocatalyst was chosen for probing the effect of high-spin Co^{2+} and S vacancies on the eNRR performance.

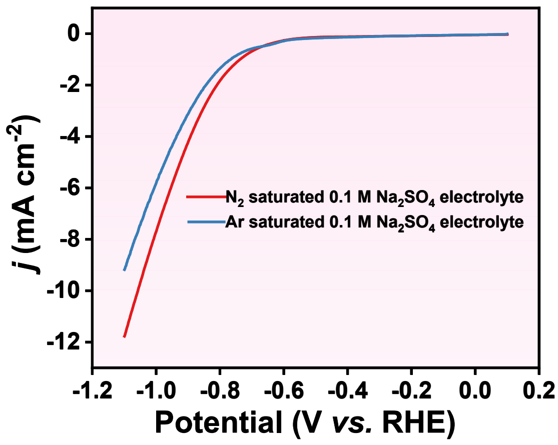

Figure S18 LSV polarization curves of Co_9S_8/Nb_2CT_x-P in an N_2- and Ar-saturated 0.1 M Na_2SO_4.

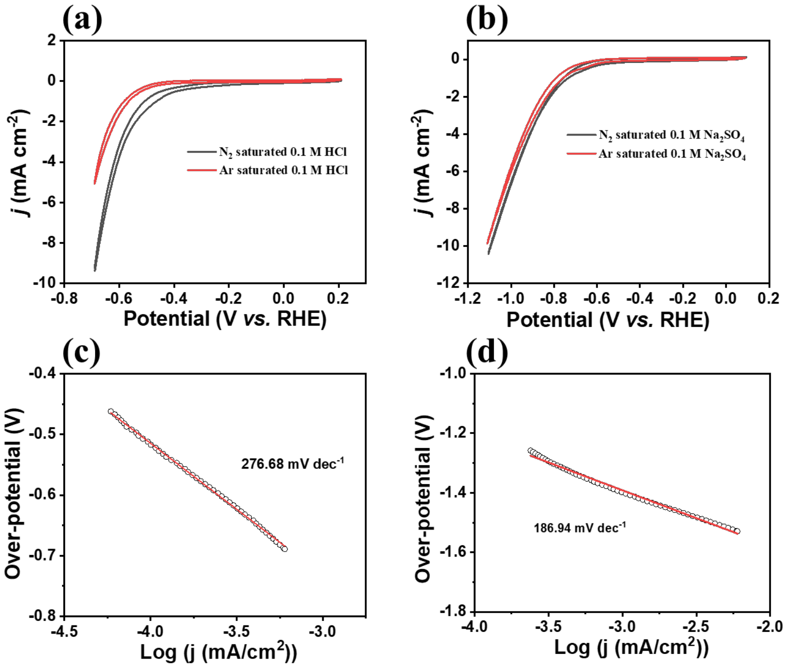

Figure S19 CV curves of Co_9S_8/Nb_2CT_x-P in Ar and N_2 saturated electrolyte, (a) 0.1 M HCl and (b) 0.1 M Na_2SO_4. Tafel slopes of Co_9S_8/Nb_2CT_x-P in (c) 0.1 M HCl and (d) 0.1 M Na_2SO_4.

Discussion: he cyclic voltammetry (CV) test of the Co_9S_8/Nb_2CT_x-P were performed at potential range of -0.7 to 0.2 V versus RHE and -1.1 to 0.1 V versus RHE at a scan rate of 5 mV s^{-1} in Ar-saturated and N_2-saturated 0.1 M HCl and 0.1 M Na_2SO_4, separately (**Figures S19a** and **b**). The results showed the distinguishable current density between the CV curves of the electrocatalysts recorded by using N_2 and Ar as feeding gas in 0.1 M HCl (**Figure S19**). Similarly, the distinct difference of the current density also can be observed in 0.1 M Na_2SO_4, suggesting the fascinating NRR activity of Co_9S_8/Nb_2CT_x-P for the efficient N_2 fixation.

As shown in **Figures S19c** and **d**, the corresponding Tafel slopes of eNRR on Co_9S_8/Nb_2CT_x-P were determined to be 276.88 mV dec^{-1} and 186.94 mV dec^{-1} in 0.1

M HCl and 0.1 M Na_2SO_4, respectively. It demonstrated that the fist electron-transfer step was the rate-limiting step ($*N_2+e^-+H^+ \rightarrow *NNH$).

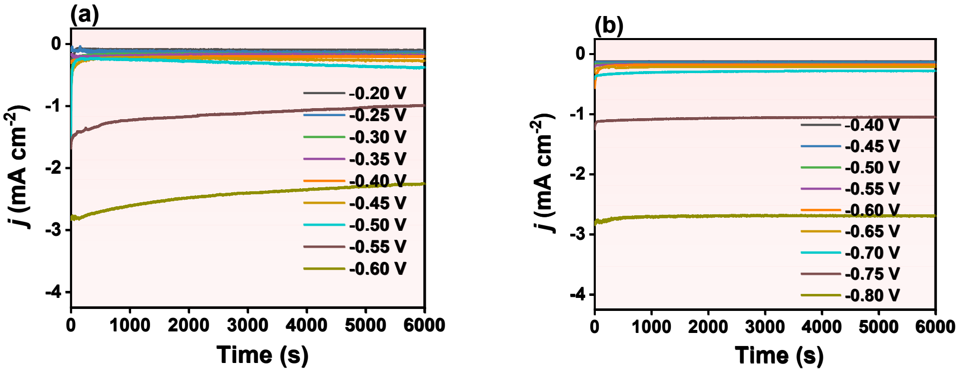

Figure S20 Chronoamperometry curves of the Co_9S_8/Nb_2CT_x-P at various potentials versus RHE in (a) 0.1 M HCl and (b) 0.1 M Na_2SO_4.

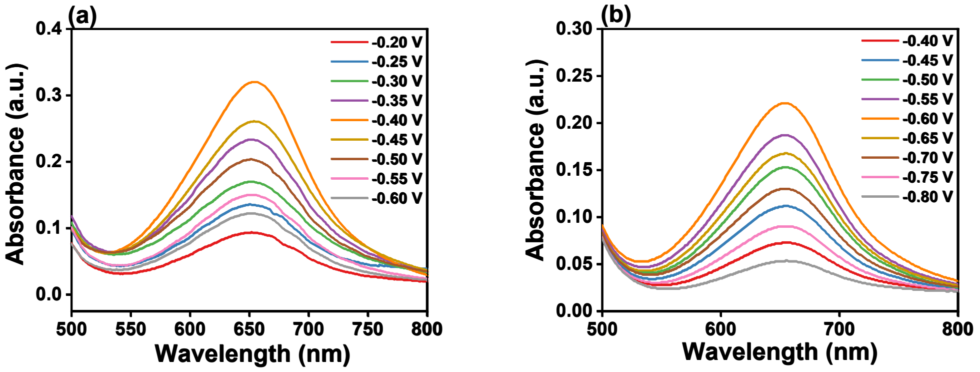

Figure S21 UV-vis absorption spectra of the (a) 0.1 M HCl and (b) 0.1 M Na_2SO_4 electrolytes stained with indophenol indicator after electrolysis at various applied potentials for 6000 s.

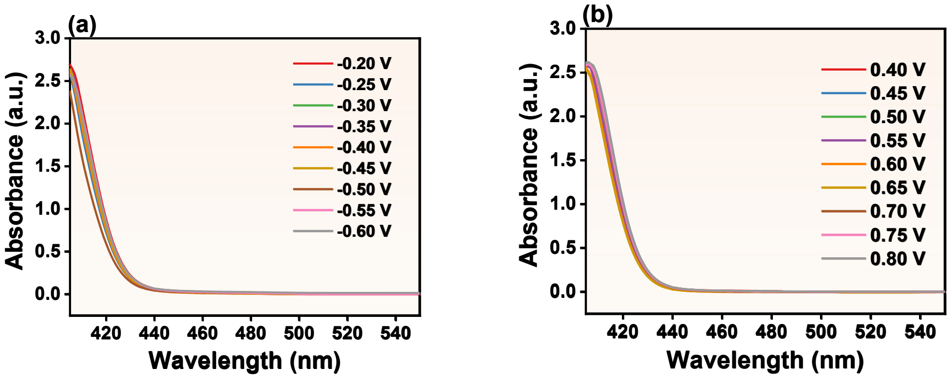

Figure S22 UV-vis absorption spectra of the (a) 0.1 M HCl and (b) 0.1 M Na_2SO_4 electrolytes stained with indophenol indicator after electrolysis at different applied potentials for 6000 s.

Table S3 Comparison of the eNRR activity of Co_9S_8/Nb_2CT_x-P with some recently reported electrocatalysts in acid electrolyte.

Catalysts	Electrolyte	NH_3 yield ($\mu g\ h^{-1}\ mg_{cat.}^{1}$)	FE (%)	Ref.
Co_4N/Co_2C@rGO	0.1 M HCl	24.12	24.97	[4]
MoFe-PC	0.1 M HCl	34.23	16.83	[5]
Ti_3C_2OH QDs	0.1 M HCl	63.49	13.88	[6]
CoS_{1-x}	0.05 M H_2SO_4	12.1	16.5	[7]
CoS_2@NC/CP	0.1 M HCl	17.45	4.6	[8]
$MnO_2-Ti_3C_2T_x$	0.1 M HCl	34.12	11.39	[9]
Co−Fe_3S_4	0.1 M HCl	37.5	17	[10]
$TiO_2/Ti_3C_2T_x$	0.1 M HCl	32.17	16.07	[11]
Zn-Co_3O_4	0.1 M HCl	22.71	11.9	[12]
BNQDs/$Ti_3C_2T_x$	0.1 M HCl	52.83	19.1	[13]
MXene/TiFeOx-700	0.05 M H_2SO_4	21.9	25.4	[14]
FeCoMOF-P_2W_{18}	0.1 M HCl	47.04	31.56	[15]
Co_9S_8/Nb_2CT_x-P	**0.1 M HCl**	**62.52**	**30.38**	**This work**

Table S4 Comparison of eNRR activity of Co_9S_8/Nb_2CT_x-P with some recently reported electrocatalysts in neutral electrolyte.

Catalysts	Electrolyte	NH_3 yield ($\mu g\ h^{-1}\ mg_{cat.}^{-1}$)	FE (%)	Ref.
ZnO-CoS QD	0.1 M Na_2SO_4	33.03	11.7	[16]
O-CoP/CNT@G	0.1 M Na_2SO_4	39.58	19.4	[17]
Co-doped MoS_{2-x}	0.01 M Na_2SO_4	10.71	10	[18]
Ni/Zn-NPC	0.1 M Na_2SO_4	22.68	1.24	[19]
boron-doped TiO_2	0.1 M Na_2SO_4	14.4	3.4	[20]
FeCoOOH HNCs	0.1 M Na_2SO_4	16.8	14.7	[21]
Au_1Co_1 alloy@GO	0.5 M K_2SO_4	36.68	22.08	[22]
C@$CoFe_2O_{4-x}$	0.1 M Na_2SO_4	30.97	11.65	[23]
S-CNS	0.1 M Na_2SO_4	19.07	7.47	[24]
Sb_2S_3@SnO_2	0.1 M Na_2SO_4	22	15.1	[25]
FeS@MoS_2/CFC	0.1 M Na_2SO_4	8.45	2.96	[26]
Co_9S_8/Nb_2CT_x-P	**0.1 M Na_2SO_4**	**41.48**	**21.39**	**This work**

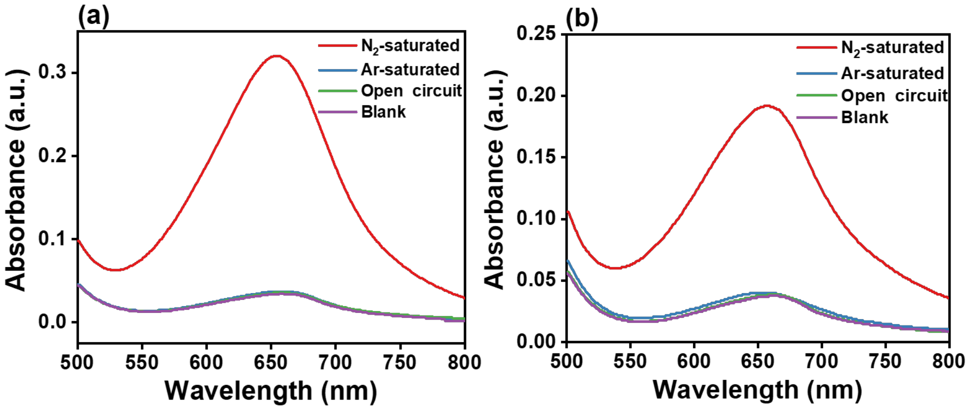

Figure S23 UV-vis absorption spectra of (a) 0.1 M HCl and (b) 0.1 M Na_2SO_4 electrolytes stained with indophenol indicator for 2 h under different conditions.

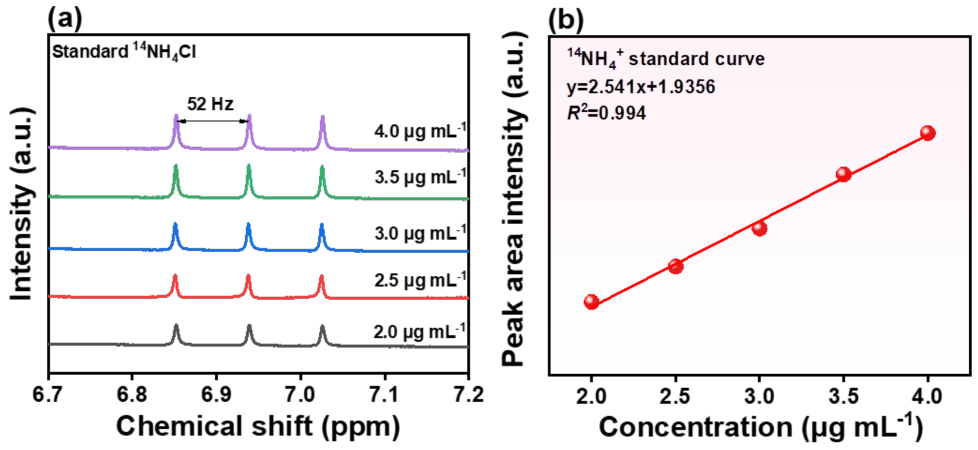

Figure S24 (a) ^{1}H NMR spectra of $^{14}NH_4^+$ standard samples in 0.1 M HCl with different concentrations and (b) the corresponding calibration curve for the evaluation of $^{14}NH_4^+$ concentration.

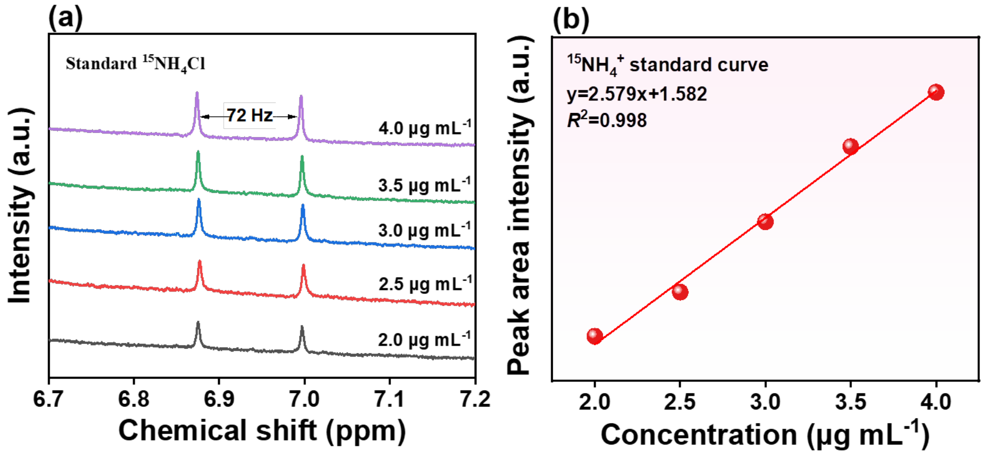

Figure S25 (a) ^{1}H NMR spectra of $^{15}NH_4^+$standard samples in 0.1 M HCl with different concentrations and (b) the corresponding calibration curve for the evaluation of $^{15}NH_4^+$ concentration.

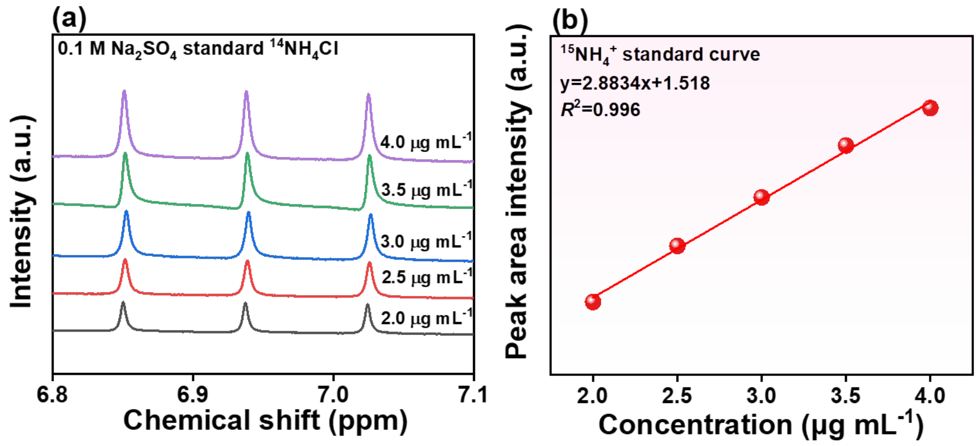

Figure S26 (a) ^{1}H NMR spectra of $^{14}NH_4^+$standard samples in 0.1 M Na_2SO_4 with different concentrations and (b) the corresponding calibration curve for the evaluation of $^{14}NH_4^+$ concentration.

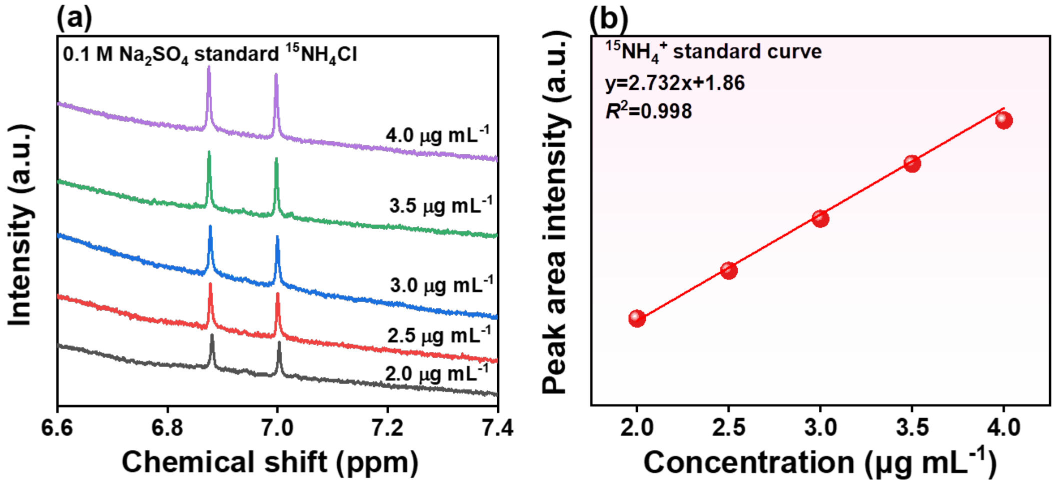

Figure S27 (a) ^{1}H NMR spectra of $^{15}NH_4^+$standard samples in 0.1 M Na_2SO_4 with different concentrations and (b) the corresponding calibration curve for the evaluation of $^{15}NH_4^+$ concentration.

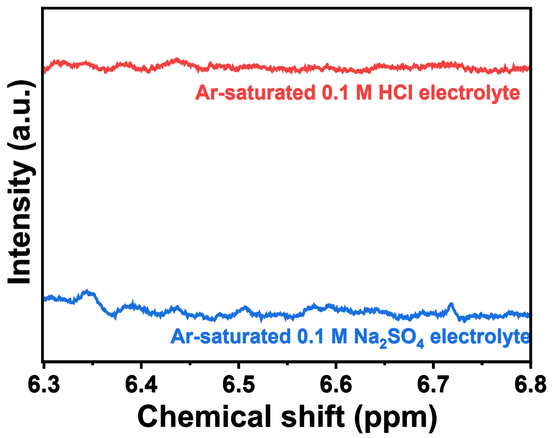

Figure S28 ^{1}H NMR spectra of 0.1 M HCl and 0.1 M Na_2SO_4 post-electrolyte for eNRR using Ar as the feeding gas.

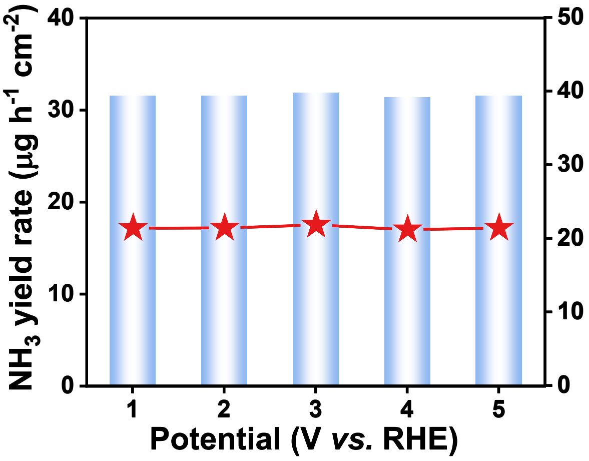

Figure S29 Stability test of Co_9S_8/Nb_2CT_x-P during the repeated eNRR for five cycles at -0.6 V versus RHE in 0.1 M Na_2SO_4.

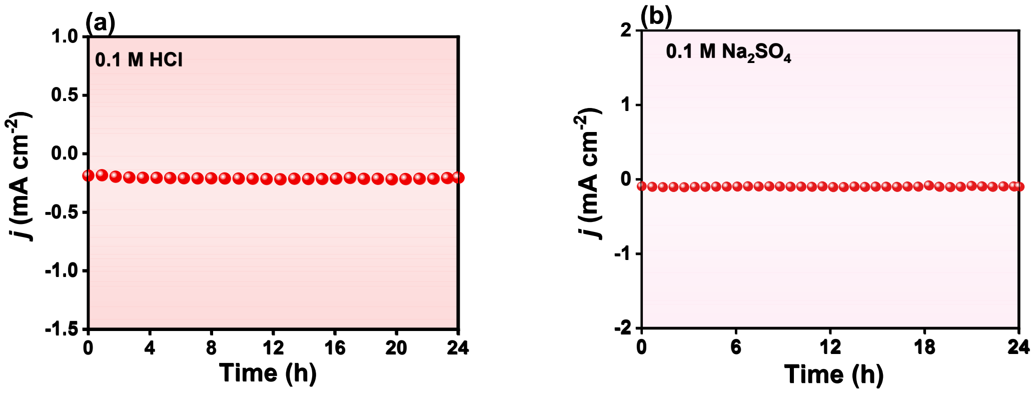

Figure S30 Chronoamperometry tests of Co_9S_8/Nb_2CT_x-P for 24 h in (a) 0.1 M HCl and (b) 0.1 M Na_2SO_4.

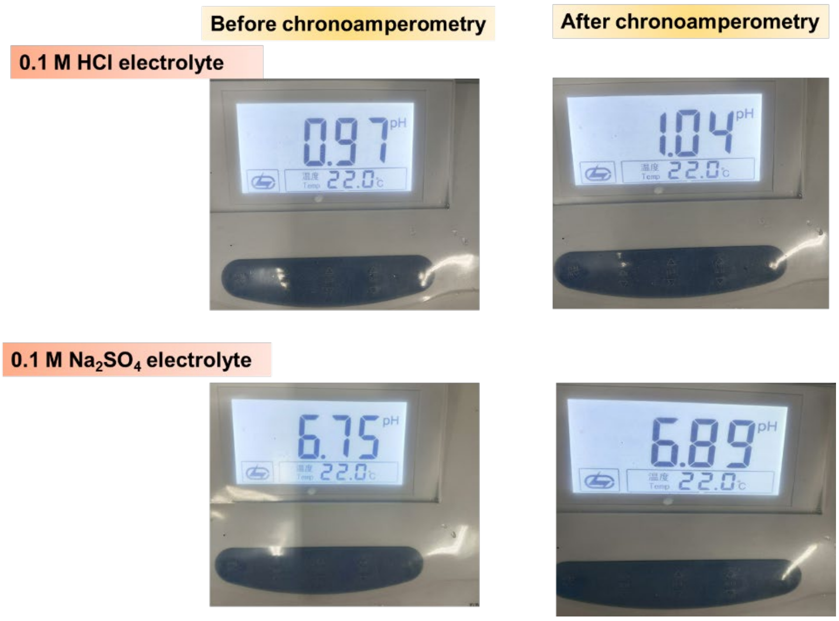

Figure S31 The pH of 0.1 M HCl and 0.1 M Na_2SO_4 catholyte before and after chronoamperometry

Discussion: As shown in **Figure S31**, only the negligible distinction in the pH before and after the eNRR was observed (in 0.1 M HCl catholyte, the as prepared electrolyte: pH value of 0.97, the post-electrolyte: pH value of 1.04; In 0.1 M Na_2SO_4 catholyte, the as prepared electrolyte: pH value of 6.75, the post-electrolyte: pH value of 6.89). The minor change in the pH value was attributed to the consumption of hydroxyl ions due to the oxygen evolution reaction in the anode chamber. It causes protons to migrate to the cathode chamber driven by the concentration difference and external applied voltage. Therefore, the pH value of electrolyte does not change significantly, even with the generation of NH_4^+.

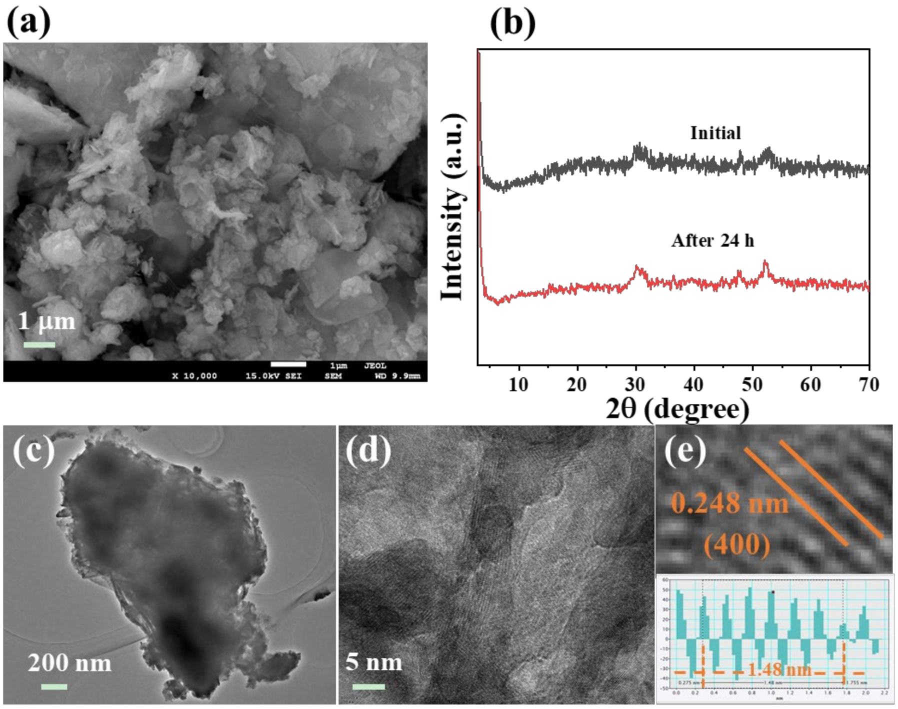

Figure S32 (a) XRD pattern, (b) SEM, (c) TEM, and (d, e) HR-TEM images of Co_9S_8/Nb_2CT_x-P after the chronoamperometry tests for 24 h in 0.1 M HCl.

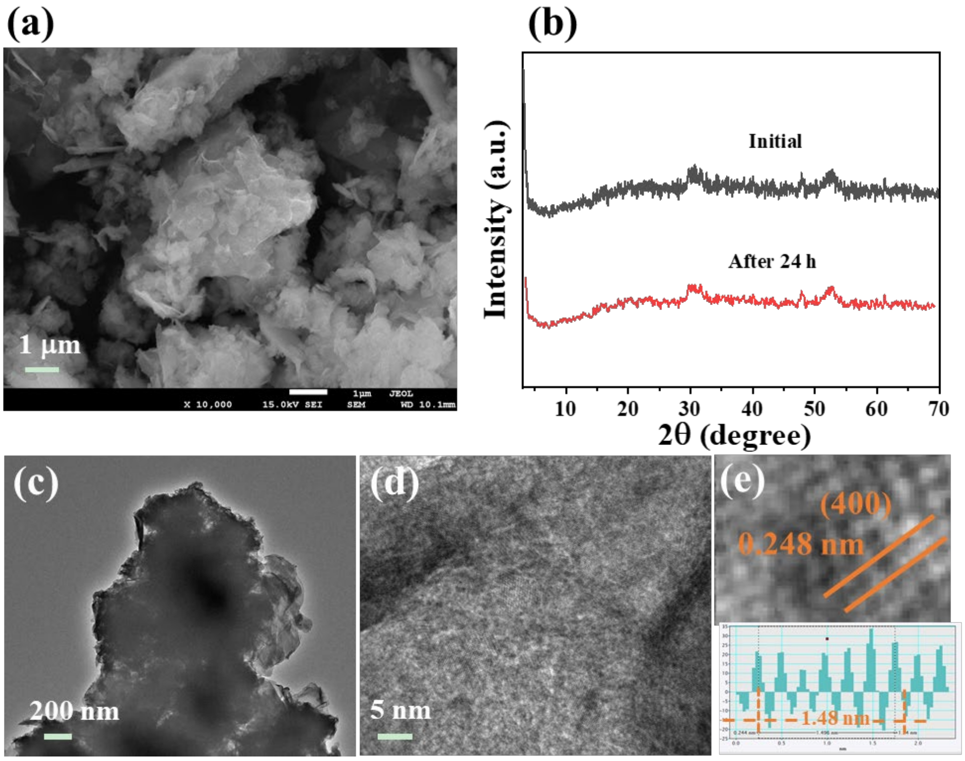

Figure S33 (a) XRD pattern, (b) SEM, (c) TEM, and (d, e) HR-TEM images of Co_9S_8/Nb_2CT_x-P after the chronoamperometry tests for 24 h in 0.1 M Na_2SO_4.

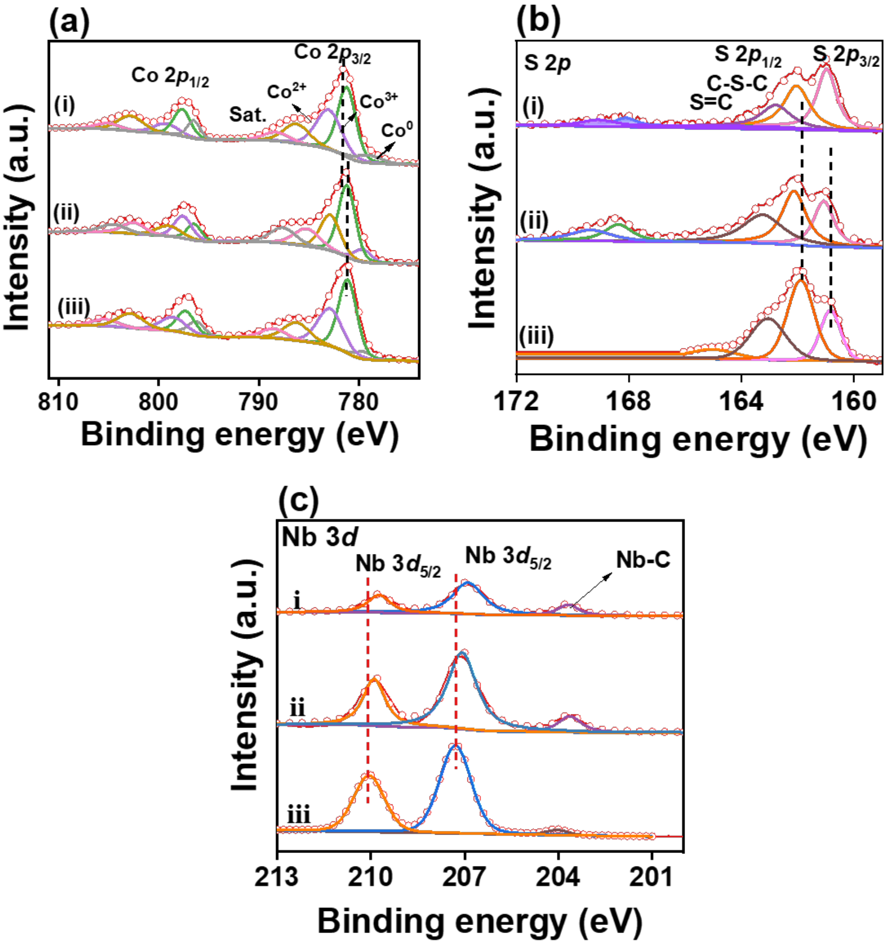

Figure S34 (a) Co 2*p*, (b) S 2*p* and (c) Nb 3*d* in the (i) used Co_9S_8/Nb_2CT_x-P in 0.1 M HCl, (ii) 0.1 M Na_2SO_4, and (iii) initial Co_9S_8/Nb_2CT_x-P.

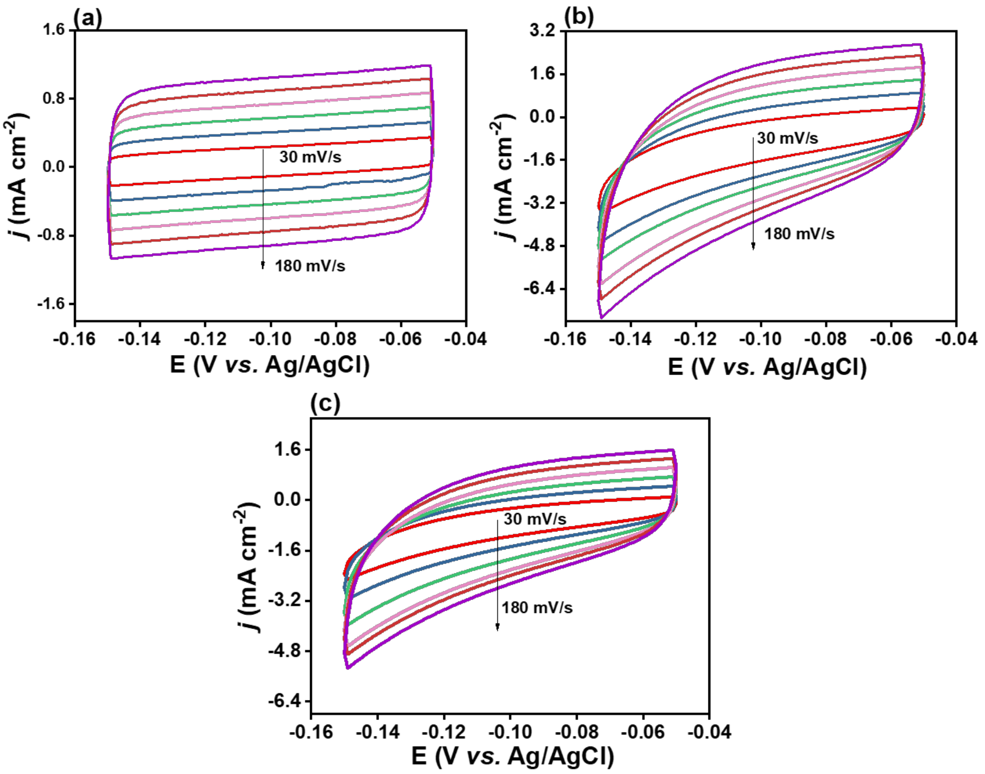

Figure S35 Cyclic voltammetry curves 0.1 M HCl of (a) Co_9S_8, (b) Co_9S_8/Nb_2CT_x and (c) Co_9S_8/Nb_2CT_x-P.

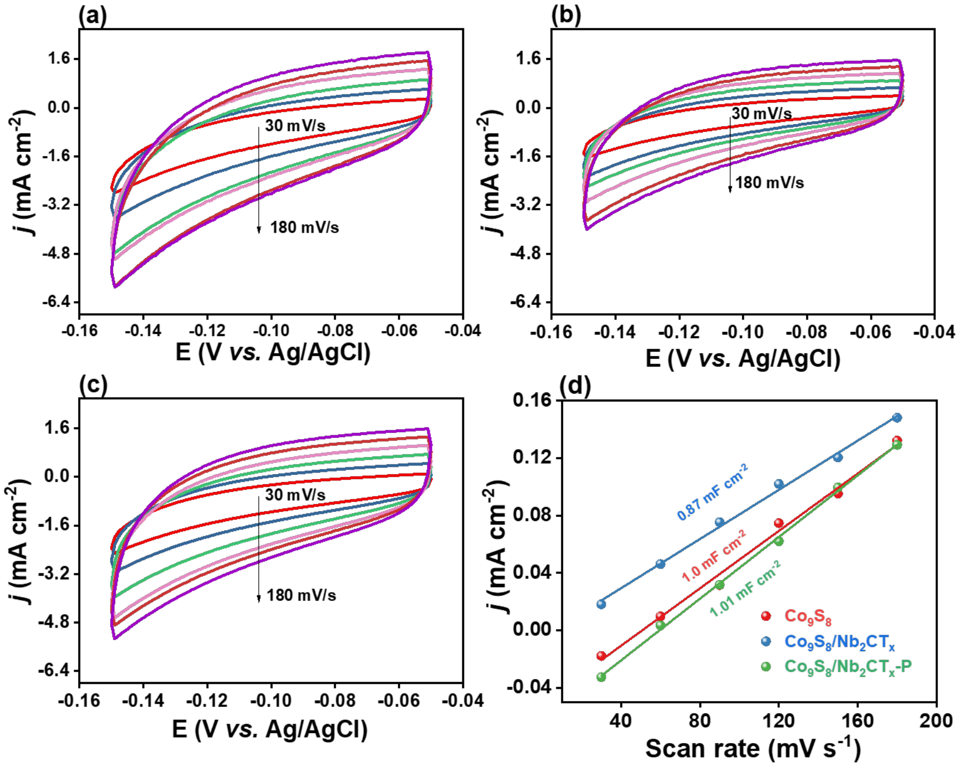

Figure S36 Cyclic voltammetry curves in 0.1 M Na_2SO_4 of (a) Co_9S_8, (b) Co_9S_8/Nb_2CT_x and (c) Co_9S_8/Nb_2CT_x-P. (d) The corresponding electrochemical double layer capacitances.

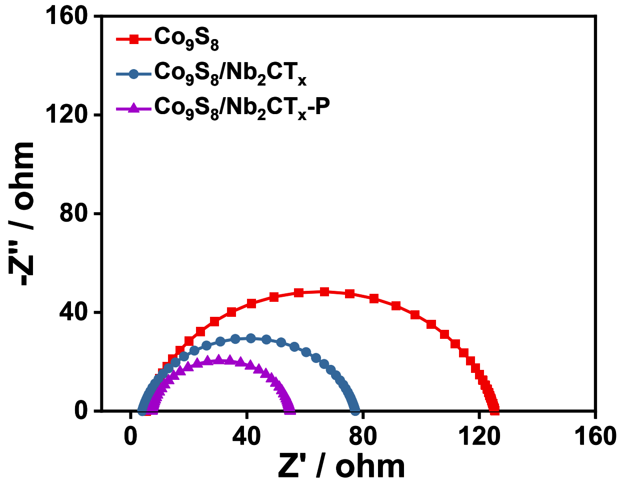

Figure S37 Nyquist plots of Co_9S_8, Co_9S_8/Nb_2CT_x and Co_9S_8/Nb_2CT_x-P at -0.6 V versus RHE in 0.1 M Na_2SO_4.

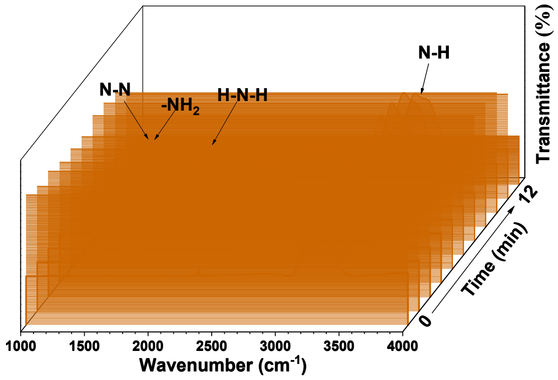

Figure S38 Electrochemical in situ ATR-FTIR spectra on Co_9S_8/Nb_2CT_x-P during NRR in 0.1 M Na_2SO_4.

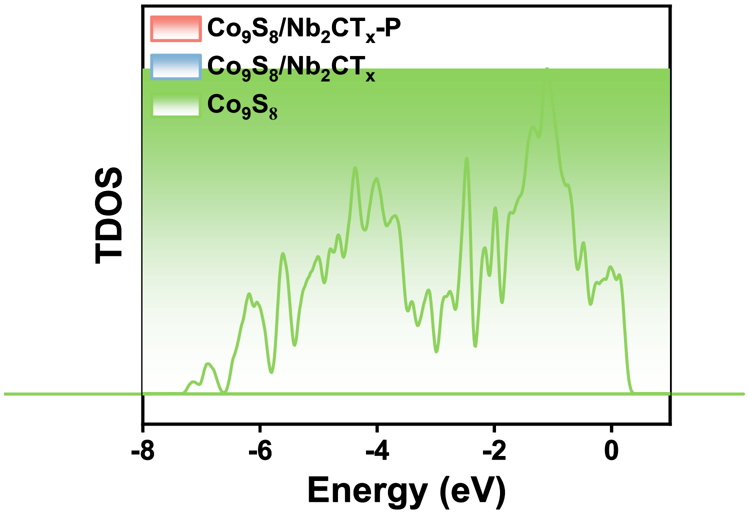

Figure S39 The projected density of states of Co 3*d* orbitals for Co_9S_8, Co_9S_8/Nb_2CT_x and Co_9S_8/Nb_2CT_x-P.

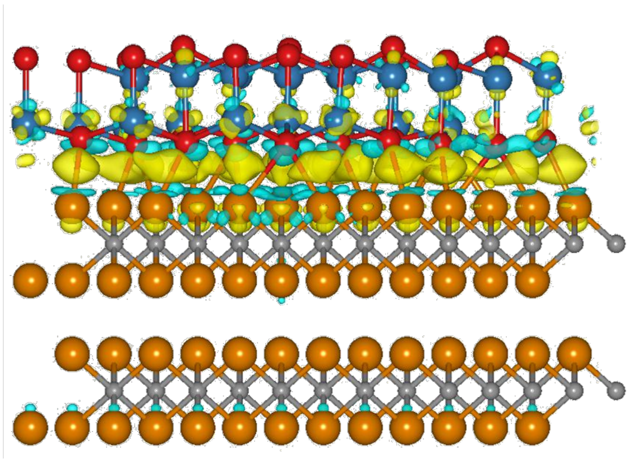

Figure S40 Calculated charge transfer for Co_9S_8/Nb_2CT_x-P (red sphere: S, blue sphere: Co, brown sphere: Nb, and gray sphere: C. Yellow and cyan-colored iso-surfaces show electron gain and loss, respectively).

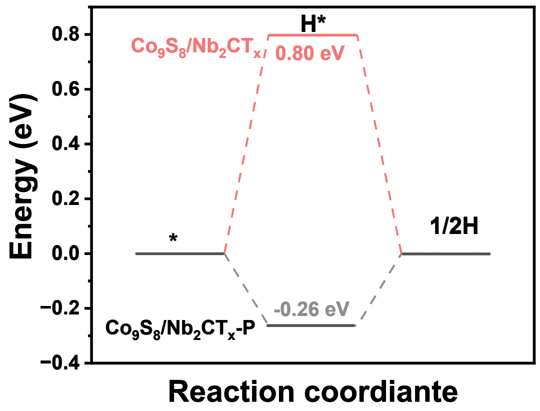

Figure S41 Gibbs free energy changed for hydrogen atom at the Co_9S_8/Nb_2CT_x and Co_9S_8/Nb_2CT_x models.

References

[1] L. Gao, C. Ma, S. Wei, A. V. Kuklin, H. Zhang, H. ågren, *ACS Nano* **2021**, *15*, 954.

[2] O. Mashtalir, M. R. Lukatskaya, M. Zhao, M. W. Barsoum, Y. Gogotsi, *Adv. Mater.* **2015**, *27*, 3501.

[3] Q. Xu, M. Xu, C. Lin, Q. Zhao, R. Zhang, X. Dong, Y. Zhang, S. Tian, Y. Tian, Z. Xia, *Adv. Sci.* **2019**, *6*, 1902043.

[4] H. Qiao, J. Yu, J. Lu, H. Bai, H. Liu, J. Hu, H. Huang, B. Wen, *ACS Sustain. Chem. Eng.* **2021**, *9*, 1373.

[5] S. Chen, H. Jang, J. Wang, Q. Qin, X. Liu, J. Cho, *J. Mater. Chem. A* **2020**, *8*, 2099.

[6] Z. Jin, C. Liu, Z. Liu, J. Han, Y. Fang, Y. Han, Y. Niu, Y. Wu, C. Sun, Y. Xu, *Adv. Energy Mater.* **2020**, *10*, 2000797.

[7] C. Li, R. Xu, S. Ma, Y. Xie, K. Qu, H. Bao, W. Cai, Z. Yang, *Chem. Eng. J.* **2021**, *415*, 129018.

[8] P. Wei, H. Xie, X. Zhu, R. Zhao, L. Ji, X. Tong, Y. Luo, G. Cui, Z. Wang, X. Sun, *ACS Sustain. Chem. Eng.* **2020**, *8*, 29.

[9] W. Kong, F. F. Gong, Q. Zhou, G. Yu, L. Ji, X. Sun, A. M. Asiri, T. Wang, Y. Luo, Y. Xu, *J. Mater. Chem. A* **2019**, *7*, 18823.

[10] X. Chen, H. Yin, X. Yang, W. Zhang, D. Xiao, Z. Lu, Y. Zhang, P. Zhang, *Inorg. Chem.* **2022**, *61*, 20123.

[11] Y. Fang, Z. Liu, J. Han, Z. Jin, Y. Han, F. Wang, Y. Niu, Y. Wu, Y. Xu, *Adv. Energy Mater.* **2019**, *9*, 1803406.

[12] L. Wen, X. Li, R. Zhang, H. Liang, Q. Zhang, C. Su, Y. Zeng, *ACS Appl. Mater.*

Interfaces **2021**, *13*, 14181.

[13] K. Chu, X. Li, Y. Tian, Q. Li, Y. Guo, *Energ. Environ. Mater.* **2022**, *5*, 1303.

[14] X. Zhao, G. Hu, G. Chen, H. Zhang, S. Zhang, H. Wang, *Adv. Mater.* **2021**, *33*, 2007650.

[15] M. Yang, X. Wang, C. J. Gómez-García, Z. Jin, J. Xin, X. Cao, H. Ma, H. Pang, L. Tan, G. Yang, Y. Kan, *Adv. Funct. Mater.* **2023**, *33*, 2214495.

[16] Y. Ji, Q. Hu, M. Yang, X. Liu, *J. Mater. Chem. A* **2023**, *11*, 25247.

[17] Q. Meng, Y. Hou, F. Yang, C. Cao, Z. Zou, J. Luo, W. Zhou, Z. Tong, S. Chen, S. Zhou, J. Wang, S. Deng, *Appl. Catal. B: Environ.* **2022**, *303*, 120874.

[18] Y. Tseng, K. Hsiao, C. Chi, M. Lu, *Mater. Today Energy* **2023**, *38*, 101425.

[19] P. Deng, Y. Liu, Y. Liu, Y. Li, R. Wu, L. Meng, K. Liang, Y. Gan, F. Qiao, N. Liu, Z. Kang, H. Li, *ACS Appl. Mater. Interfaces* **2023**, *15*, 44809.

[20] Y. Wang, K. Jia, Q. Pan, Y. Xu, Q. Liu, G. Cui, X. Guo, X. Sun, *ACS Sustain. Chem. Eng.* **2019**, *7*, 117.

[21] X. Han, C. Liu, Y. Tang, Q. Meng, W. Zhou, S. Chen, S. Deng, J. Wang, *J. Mater. Chem. A* **2023**, *11*, 14424.

[22] Q. Wang, G. Zheng, S. Hao, X. Liu, J. Zheng, Y. Wang, Z. Su, N. Xu, Y. He, L. Lei, X. Zhang, *ACS Sustain. Chem. Eng.* **2020**, *8*, 44.

[23] C. Wang, L. Gu, S. Qiu, J. Gao, Y. Zhang, K. Wang, J. Zou, P. Zuo, X. Zhu, *Appl. Catal. B: Environ.* **2021**, *297*, 120452.

[24] L. Xia, X. Wu, Y. Wang, Z. Niu, Q. Liu, T. Li, X. Shi, A. M. Asiri, X. Sun, *Small Methods* **2019**, *3*, 1800251.

[25] Di Li, X. Chen, Y. Liu, J. Yu, B. Ding, *Chem. Commun.* **2019**, *55*, 13892.

[26] Y. Guo, Z. Yao, B. J. J. Timmer, X. Sheng, L. Fan, Y. Li, F. Zhang, L. Sun, *Nano Energy* **2019**, *62*, 282.
